# A Three in One Strategy to Achieve Zirconium Doping, Boron Doping, and Interfacial Coating for Stable LiNi_0.8_Co_0.1_Mn_0.1_O_2_ Cathode

**DOI:** 10.1002/advs.202001809

**Published:** 2020-11-27

**Authors:** Ze Feng, Ranjusha Rajagopalan, Shan Zhang, Dan Sun, Yougen Tang, Yu Ren, Haiyan Wang

**Affiliations:** ^1^ Hunan Provincial Key Laboratory of Chemical Power Sources Hunan Provincial Key Laboratory of Efficient and Clean Utilization of Manganese Resources College of Chemistry and Chemical Engineering Central South University Changsha 410083 P. R. China; ^2^ TEC Materials Development Team Tianmu Lake Institute of Advanced Energy Storage Technologies Changzhou 213300 P. R. China

**Keywords:** LiNi_0.8_Co_0.1_Mn_0.1_O_2_, oxygen vacancies, structural stability, thermal stability, ZrB_2_

## Abstract

LiNi_0.8_Co_0.1_Mn_0.1_O_2_ cathodes suffer from severe bulk structural and interfacial degradation during battery operation. To address these issues, a three in one strategy using ZrB_2_ as the dopant is proposed for constructing a stable Ni‐rich cathode. In this strategy, Zr and B are doped into the bulk of LiNi_0.8_Co_0.1_Mn_0.1_O_2_, respectively, which is beneficial to stabilize the crystal structure and mitigate the microcracks. Meanwhile, during the high‐temperature calcination, some of the remaining Zr at the surface combined with the surface lithium source to form lithium zirconium coatings, which physically protect the surface and suppress the interfacial phase transition upon cycling. Thus, the 0.2 mol% ZrB_2_‐LiNi_0.8_Co_0.1_Mn_0.1_O_2_ cathode delivers a discharge capacity of 183.1 mAh g^−1^ after 100 cycles at 50 °C (1C, 3.0–4.3 V), with an outstanding capacity retention of 88.1%. The cycling stability improvement is more obvious when the cut‐off voltage increased to 4.4 V. Density functional theory confirms that the superior structural stability and excellent thermal stability are attributed to the higher exchange energy of Li/Ni exchange and the higher formation energy of oxygen vacancies by ZrB_2_ doping. The present work offers a three in one strategy to simultaneously stabilize the crystal structure and surface for the Ni‐rich cathode via a facile preparation process.

## Graphical abstract

A three in one strategy using ZrB_2_ dopant modification is proposed for constructing stable LiNi_0.8_Co_0.1_Mn_0.1_O_2_ cathodes. The structural and interfacial stability of the material can be improved owing to the Zr and B doping in the bulk and the formation of lithium zirconium oxide coating layer. This strategy provides rational guidance for designing high‐performance Ni‐rich cathode materials.

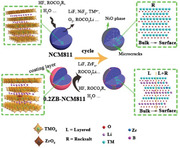

## 1 Introduction

Ni‐rich ternary material, LiNi_0.8_Co_0.1_Mn_0.1_O_2_ (NCM811), has attracted considerable attention as a promising cathode due to its high specific discharge capacity (>200 mAh g^−1^) and low cost, which is considered to be the ideal features to overcome the drawback of short‐recharge mileage in electric vehicles.^[^ ^1^ ^]^ However, because of its unstable crystal structure and interface, this kind of material has suffered from severe capacity fading during prolonged cycling.^[^ ^2^ ^]^ For instance, a series of phase transitions can be occurred accompanied by repeated Li^+^ insertion/extraction. Initially, at a low voltage, there is an insulator–metal phase transition with an O3 phase (the hexagonal structure) in R‐3m space group (where both transition metal (TM) ions and Li^+^ occupy at the octahedral sites). When charged to 4.2 V, the material undergoes a transition from O3 phase to C2/m phase (monoclinic structure with Li ions occupying 2*a* and 4*c* sites, and TM ions are placed at 2*b* and 4*c* sites) and back to O3 phase.^[^ ^3^ ^]^ Moreover, it will experience phase transition from O3 phase to O6 phase when charged to above 4.4 V.^[^ ^4^ ^]^ These phase transitions cause significant volumetric variations, lattice distortions, and Li vacancies formation as well as oxygen loss. The formation of Li vacancies can cause Ni^2+^ (has a similar radius to Li^+^) to gradually migrate to the Li^+^ positions, leading to severe cation mixing and structural degradation.^[^ ^5^ ^]^ The oxygen loss can cause the dissolution of the TM, resulting in severe voltage decay and safety issue. Intragranular cracking, frequently derived from the volumetric variations and lattice distortions of the layered cathodes, has been proved to be a major contributor to performance decay.^[^ ^6^ ^]^ Furthermore, the attenuation of electrochemical performances is also caused by unstable interfaces.^[^ ^7^ ^]^ The integrity of cathode–electrolyte‐interface (CEI) also gets affected due to the detrimental side reactions at the electrode/electrolyte interface when charged to a high voltage. This can lead to new CEI formation (while reducing active Li^+^) as well as the TMs exposure to electrolyte and thereby increase resistance.^[^ ^8^ ^]^ The exposed TMs can cause an accelerated decomposition of the LiPF_6_ electrolyte into OPF_3_, LiF, and HF compounds, and the HF will severely corrode the current collector, leading to the loss of active materials and the electrochemical performance deterioration.^[^ ^9^ ^]^

In response to these problems, many strategies have been proposed to improve the structural and interfacial stability of the electrode. For example, element doping has been proved to be one of the most effective strategies for mitigating the problems associated with irreversible transition.^[^ ^10^ ^]^ Doping elements such as Al, Zr, Ti, Nd, Mo, B, and Mg have been reported.^[^ ^11^ ^]^ Liu et al.^[^ ^12^ ^]^ confirmed that the lattice expansion can be suppressed when substituting the TMs with Ti. Al has also been widely studied in the ternary cathode material due to its superior stability. The addition of Al into the TM sites endows lower cation mixing and superior cycling stability for the Ni‐rich cathode.^[^ ^13^ ^]^ Another study reported that the boron‐doped Ni‐rich material could relieve the intrinsic internal strain and suppress the microcracks, thereby improving the structural stability.^[^ ^14^ ^]^ In addition, the surface coating is also widely used for protecting the interface, and many compounds (Al_2_O_3_, ZrO_2_, Li_3_PO_4_, PANI‐PVP, and Y_2_O_3_) have been used as effective coatings.^[^ ^15^ ^]^ The purpose of using such coatings are commonly to isolate active materials from the electrolyte to suppress the side reactions, reduce the HF‐content and improve the cycling stability. Till date, the aforementioned approaches have been utilized to mitigate the structural and interfacial degradation. Note that most of these research approaches focused on either elemental doping or surface coating; hence, it is still a big challenge to achieve both structural and interfacial stability for Ni‐rich ternary materials at the same time due to process complexities.

Herein, we proposed a three in one strategy using ZrB_2_ dopant for constructing stable LiNi_0.8_Co_0.1_Mn_0.1_O_2_ cathode. This strategy could achieve both Zr and B doping in the bulk phase, and also some of the remained Zr on the surface combined with the surface lithium source to form protective layers during the sintering process. That is, three functions including Zr doping, B doping, and interfacial coating could be well performed via a one‐step approach in this work. The as‐prepared LiNi_0.8_Co_0.1_Mn_0.1_O_2_ cathode delivers much higher electrochemical properties at room and elevated temperature. More importantly, this electrode also shows superior cycling stability at higher cut‐off voltage to 4.4 V, by which higher energy density of LIBs could be achieved. The reasons why the treated sample exhibited superior electrochemical properties have been well explained by experimental characterizations and density functional theory (DFT) calculation.

## 2 Results and Discussion

**Figure 1:**
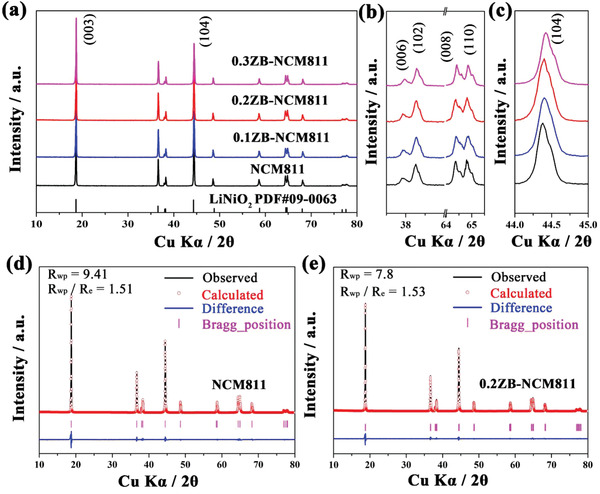
a) XRD patterns of all samples; b,c) The corresponding enlarged view of 34–68° and 44–45°. d,e) Rietveld refinement results of NCM811 and 0.2ZB‐NCM811.

The crystal structures of NCM811, 0.1ZB‐NCM811, 0.2ZB‐NCM811, and 0.3ZB‐NCM811 have been characterized by X‐ray diffraction (XRD) patterns and the results are shown in **Figure** **1**. All samples show high crystallinity with a rhombohedral crystal structure (R‐3m space group), as well as no obvious impurity phases, and the clear peak splitting of (006)/(102) and (108)/(110) (Figure 1b) indicate well‐defined layered structure.^[^ ^16^ ^]^ In addition, the (104) peak shifts to the right slightly with increasing ZrB_2_ content in Figure 1c, while the (104) peak shifts to the left in the sample with only Zr doping according to the previous report.^[^ ^17^ ^]^ This opposite result could be attributed to co‐incorporation of the zirconium and boron into the NCM811 lattice. It has been well confirmed by other groups that the B element mainly occupied the tetrahedral interstitial sites in the oxygen layer due to the smaller ionic radius,^[^ ^14^ ^]^ and the Zr substitutes the TM site to form a more stable structure.^[^ ^18^ ^]^ Rietveld refinement results of prepared samples are displayed in Figure 1d‐,e and Figure S1 of the Supporting Information, and the relative lattice parameters are calculated and listed in Table S1 of the Supporting Information. The results indicate that all the samples exhibit low cation mixing and well‐developed R‐3m structure due to the values of *I*_003_/*I*_104_ and c/a which are higher than 1.21 and 4.94, respectively.^[^ ^11b^ ^]^ Furthermore, the unit cell expansion is also observed in modified samples as the lattice parameters of *a* and *c* increase after doping. This could be attributed to the Zr and B doping into the TM layer and oxygen layer, respectively. Zr^4+^ has a larger radius with 0.072 nm than those of the Mn^4+^ (0.053 nm), Ni^2+^ (0.069 nm), and Co^3+^ (0.0685 nm), which enlarges the unit cell and improves the lithium‐ion diffusion ability in the bulk phase.^[^ ^19^ ^]^ Furthermore, the B atoms at the tetrahedral interstitial sites in the oxygen layer also can enlarge the lattice parameters of *a* and *c*.^[^ ^20^ ^]^ Interestingly, based on the analyses of *I*_003_/*I*_104_ values and Li^+^/Ni^2+^ mixing, the 0.2% ZrB_2_ doping shows the best positive effect in suppressing the cation disorder.^[^ ^21^ ^]^

**Figure 2:**
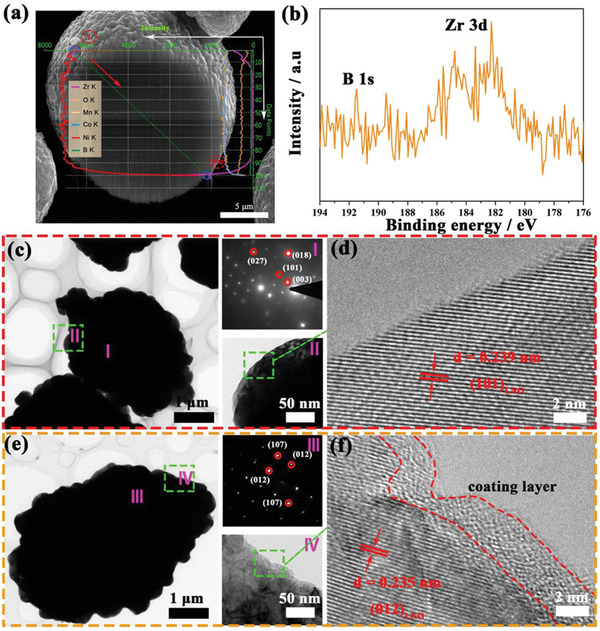
a) Energy dispersive X‐ray (EDX) analysis image on the cross section of the 0.2ZB‐NCM811; b) XPS spectra of Zr and B of the 0.2ZB‐NCM811; c,e) TEM images of NCM811 and 0.2ZB‐NCM811; d,f) The corresponding HR‐TEM images.

Morphologies of the prepared precursor and samples were characterized by SEM as shown in Figure S2, Supporting Information. The samples display a spheroidal morphology with the particle size of ≈10 µm (Figure S2a–c, Supporting Information). The particles of both NCM811 and 0.2ZB‐NCM811 comprise of densely aggregated primary particles as observed from the corresponding high‐magnification SEM images, and it shows a similar morphology due to the low doping of boron.^[^ ^14^ ^]^ To clarify the Zr and B distribution in the 0.2ZB‐NCM811, the elemental mapping images of SEM and cross‐sectional SEM are performed (Figure S2d–i, Supporting Information). Evidently, the Zr element is well distributed throughout the material, not only in the bulk but also at the interfaces of 0.2ZB‐NCM811 sample (Figure S2e,h, Supporting Information), that is, in addition to the doping into the bulk, a portion of remained Zr on the surface can react with the lithium to form a coating layer onto the sample during the sintering.^[^ ^17^ ^]^ However, the B element is mainly detected in the bulk (Figure S2f,i, Supporting Information), and the uniform distribution of B in the particle interior is also achieved as shown in Figure S2i, Supporting Information, demonstrating that the B is successfully doped into the crystal lattice. Moreover, the energy dispersive X‐ray analysis on the cross section of the 0.2ZB‐NCM811 is shown in **Figure** **2**a. The result shows that a large amount of Zr remained on the surface of the material, while the B element is mainly doped in the bulk. It should be attributed to the different ionic radius and the different migration energies between Zr and B from the surface to the interior. The B trends to dope into the bulk due to its smaller ionic radius and the lower migration energy of B. This is similar to the Zr and Al co‐doping in the Ni‐rich cathode.^[^ ^22^ ^]^ In order to further study the surface chemical properties of the pristine NCM811 and 0.2ZB‐NCM811, the X‐ray photoelectron spectra (XPS) was employed and the results are shown in Figure 2b and Figure S3, Supporting Information. The characteristic peak of Zr is detected in Figure 2b, while that of B is difficult to detect, revealing that the Zr element exists on the surface of the material and the B is mainly doped in the bulk. It is worth noting that the binding energy of Ni, Co, and Mn have some weak negative shift after ZrB_2_ treatment (Figure S3b–d, Supporting Information), which may come from the concentration effect of TM ion. The accumulation of Zr on the surface reduces the content of TM ion.^[^ ^19b^ ^]^

Figure 2c–f provides the TEM and selected area electron diffraction (SAED) images of NCM811 and 0.2ZB‐NCM811 samples. The TEM and SAED images indicate that all the samples exhibit a crystalline structure.^[^ ^23^ ^]^ High‐resolution transmission electron microscopy (HR‐TEM) images show the formation of the coating layer with a thickness of ≈2 nm (Figure 2f; Figure S4a,b, Supporting Information) after being modified by ZrB_2_. This coating layer (lithium zirconium oxide) was formed by combing the Zr which remained on the surface with the surface lithium source during high sintering temperature. It was confirmed by Gao et al. that Zr could react with the residual lithium and form lithium zirconates when using Zr salt as the dopant.^[^ ^17^ ^]^ Besides, the planes of (101) and (012) are detected, corresponding to the interplanar spacing of 0.239 and 0.235 nm, respectively, which confirms the successful synthesis of well‐crystallized Ni‐rich cathode. According to the above analysis, we can conclude that B and Zr have concurrently doped into the bulk lattice though a simple preparation process, which is beneficial for stabilizing the bulk structure of the material. Meanwhile, some of the remained Zr combined with the surface lithium source to form lithium zirconium oxide layer during the high‐temperature calcination, which may help to protect the interface of the material.

**Figure 3:**
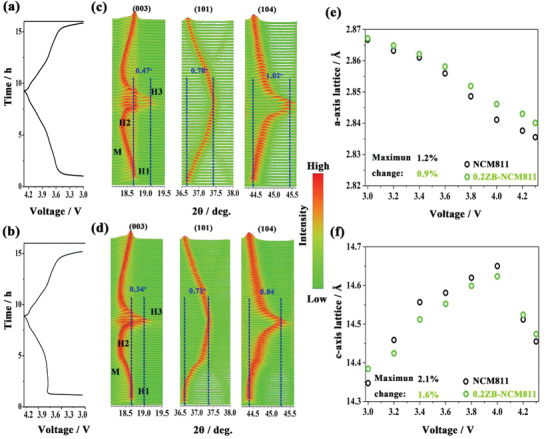
In situ XRD measurements for a) NCM811 and b) 0.2ZB‐NCM811 cathodes during the first charge–discharge process (between 3.0 and 4.3 V) at 0.1C; c,d) the corresponding shifts of (003), (101), and (104) reflections; Corresponding calculated lattice parameters along e) *a*‐axis and f) *c*‐axis of the two samples on charge as a function of the cell voltage.

To get insight into the structural stability of the modified sample, in situ XRD measurements on the NCM811 and 0.2ZB‐NCM811 cells were performed and the result is shown in **Figure** **3**. Apparently, both cathodes exhibit a normal lithiation/delithiation process during the cycle (Figure 3a,b). The (003) and (101) reflection of the Ni‐rich cathode are related to the variation of the *c*‐axis and *a*‐axis, respectively.^[^ ^24^ ^]^ As shown in Figure 3c,d, both samples experienced H1 → M, M → H2, and H2 → H3 phases transition. Meanwhile, the (003) reflection of both samples moved to a lower angle first during the charging process and then shifted sharply to a higher angle. This change process can cause the expanding and shrinking for the lattice parameter of *c*, which is mainly ascribed to the weaker screening effect of depleted Li layers and the enhanced repulsion between the oxygen layers during the Li ions deintercalation.^[^ ^4^, ^15^ ^]^ In comparison with the (003) reflection, the (110) reflection shifts are more simple and vary upon charging, suggesting a roughly continuous decline in lattice parameter *a*.^[^ ^25^ ^]^ Based on in situ XRD data, the offsets of (003), (101), and (104) peaks for the pristine sample are 0.47°, 0.78°, and 1.02°, respectively, while the 0.2ZB‐NCM811 shows lower values with 0.37°, 0.71°, and 0.84°, confirming that the more stable planes of (003), (101), and (104) for doped sample.^[^ ^26^ ^]^ In addition, to quantitatively assess the extent of lattice parameters during Li extraction, we have tracked the change in *c*‐axis and *a*‐axis lattice parameters during the charging process. As shown in Figure 3e,f, when charging from 3.0 to 4.3 V, the lattice parameters variations of *a*‐axis and *c*‐axis for NCM811 are 1.2% and 2.1% respectively, while those for 0.2ZB‐NCM811 are 0.9% and 1.6%, indicating that ZrB_2_ doping can effectively suppress the lattice distortion for Ni‐rich cathode.

**Figure 4:**
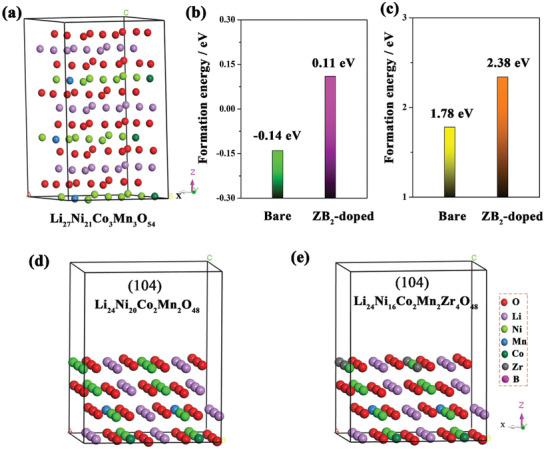
a) Structural model for Li_27_Ni_21_Mn_3_Co_3_O_54_; b,c) The exchange energy of Li/Ni exchange and the formation energy of oxygen vacancy for the bare and doped samples; d) Li_24_Ni_20_Co_2_Mn_2_O_48_ surface constructed along the (104) direction, along with a vacuum slab of 8 Å, e) Li_24_Ni_16_Co_2_Mn_2_Zr_4_O_48_ surface after the surface doping of four Zr atoms based on (d).

To provide mechanistic insights into the experimental observations, first principles calculation was used to study the different penetration energies for Zr and B during the doping process. The DFT calculations for the Ni‐rich cathode are described in detail in Supporting Information. The model and the result are shown in Figure S5 and Table S2, Supporting Information. As seen in the Figure S5, Supporting Information, the calculated migration energies from the surface to bulk for B and single B doped in Zr‐B coexist on the surface are 2.76 and 3.16 eV, which is lower than that of Zr (3.67 eV) and single Zr doping (6.41 eV), indicating that B is easier to migrate into the bulk than Zr. The migration resistance of Zr will greatly increase when Zr and B are in coexistence. This may provide a rational understanding of why partial Zr accumulates on the surface and the B accumulates in the bulk. To gain a better understanding of the improved structural stability of modified sample, we have calculated the energies of Li/Ni exchange and oxygen vacancy formation for LiNi_0.8_Co_0.1_Mn_0.1_O_2_ and doped LiNi_0.8_Co_0.1_Mn_0.1_O_2_. The structural stability and thermal stability for Ni‐rich cathode are closely related to the Li/Ni exchange and oxygen vacancy formation.^[^ ^27^ ^]^ The corresponding models for pristine and doped sample were built and are shown in **Figure** **4**a and Figure S6, Supporting Information. The exchange energy of Li/Ni exchange for the pristine sample is calculated to be −0.14 eV, and after doping with Zr and B the value is increased to 0.11 eV, indicating that there is a much higher resistance for Li/Ni exchange (Figure 4b).^[^ ^28^ ^]^ The higher Li/Ni exchange energy of the doped sample should be attributed to Zr and B co‐doping. On the one hand, Ni^2+^ migration from TM layer to Li layer is hindered due to the strong electrostatic interaction between the Ni^2+^ and Zr^4+^ ions when doping with Zr^4+^ at Ni sites.^[^ ^18^ ^]^ The Zr can maintain an optimal cation ordering and stabilize the TM layer because of its absence in the redox reaction.^[^ ^29^ ^]^ On the other hand, the strong crystallographic framework within each secondary particle is constructed by B doping because of the formation of boracic polyanions between B and O atoms.^[^ ^30^ ^]^ In addition, the formation energy of oxygen vacancy in the bulk for the doped sample is 2.38 eV, which is also much higher than that of the pristine one (1.78 eV; Figure 4c). Also, we have investigated the stability of the surface oxygen for the bare and surface doped sample. The models and the calculated results are shown in Figure 4d,e and Figure S7, Supporting Information. The formation energy of surface oxygen vacancy for Zr accumulates model is 4.35 eV, which is also higher than the bare sample model of 3.69 eV, demonstrating that Zr accumulation at the surface is beneficial for stabilizing the surface oxygen atoms. Combined with the above analysis, it can conclude that the modified sample has a more stable O^2−^ anion framework, which may due to the stronger bond energies of Zr—O (760 kJ mol^−1^) and B—O (806 kJ mol^−1^). These results reveal that the three in one strategy with ZrB_2_ treatment could greatly improve the phase and oxygen atom stability, thereby enhancing the structural and thermal stability of Ni‐rich cathode.^[^ ^31^ ^]^

**Figure 5:**
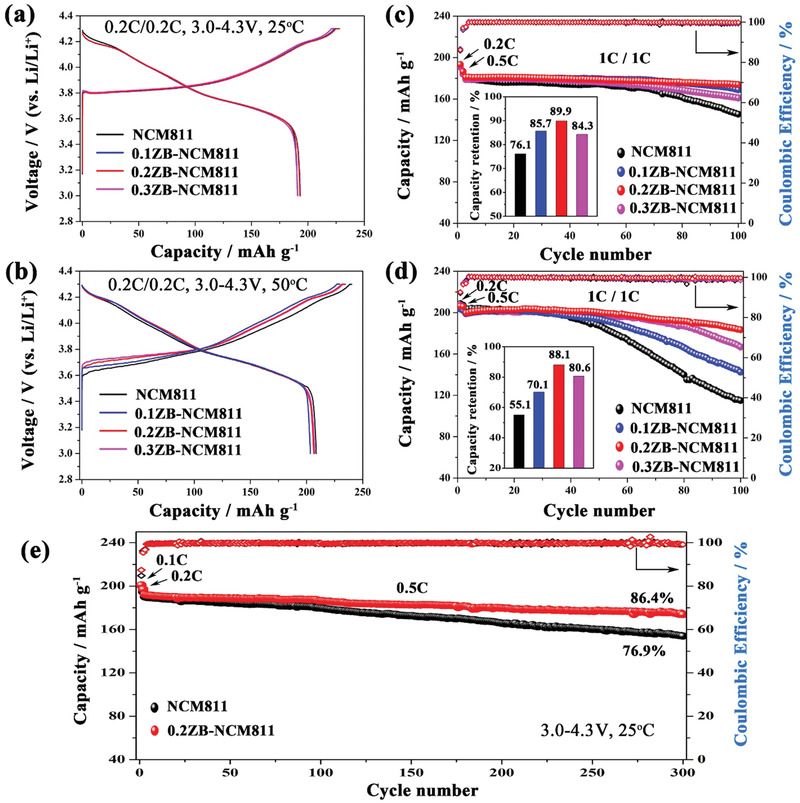
Initial charge−discharge curves at a) 25 °C and b) 50 °C and cycle performances at c) 25 °C and d) 50 °C between 3.0 and 4.3 V for NCM811, 0.1ZB‐NCM811, 0.2ZB‐NCM811, and 0.3ZB‐NCM811 electrodes; e) Galvanostatic cycling for NCM811 and 0.2ZB‐NCM811 at 0.5C for 300 cycles after two activation cycles at 0.1C and 0.2C.

Electrochemical performances of as‐prepared samples were investigated at different temperatures between 3.0 and 4.3 V. As shown in **Figure** **5**, the prepared electrodes deliver similar discharge capacity of ≈192 mAh g^−1^ at 25 °C in the initial cycle (0.2C, Figure 5a). Also, all the samples show a higher initial discharge capacity of ≈205 mAh g^−1^ when tested at an elevated temperature of 50 °C because of the higher ionic activity (0.2C, Figure 5b).^[^ ^32^ ^]^ However, the pristine NCM811 exhibits a rapid capacity decline upon cycling at 1C (Figure 5c) with a capacity retention of 76.1% after 100 cycles. This value is much lower than that of doped samples, that is, capacity retention of 85.7%, 89.9%, and 84.3% is observed for 0.1ZB‐NCM811, 0.2ZB‐NCM811, and 0.3ZB‐NCM811, respectively. Furthermore, the capacity fading becomes more severe when tested at 50 °C(Figure 5d), where the pristine NCM811 electrode could only retain 55.1% of its initial discharge capacity after 100 cycles. Interestingly, the capacity retention for the modified samples is greatly improved compared to the pristine one, and the 0.2ZB‐NCM811 shows the best electrochemical performance, delivering a higher discharge capacity of 183.1 mAh g^−1^ with a capacity retention of 88.1% for the same cycling period at elevated temperature. Meanwhile, the discharge capacity of 0.1ZB‐NCM811 and 0.3ZB‐NCM811 is 142.5 and 166.9 mAh g^−1^ and the capacity retention is 70.1% and 80.6%, respectively. These results indicate that proper ZrB_2_ treatment can greatly improve the electrochemical performance of Ni‐rich cathode.^[^ ^33^ ^]^ In addition, the long‐term cycling stability of NCM811 and 0.2ZB‐NCM811 were tested at 0.5C between 3.0 and 4.3 V (25 °C, Figure 5e). Similarly, the 0.2ZB‐NCM811 provides a higher discharge capacity of 173.8 mAh g^−1^ even after 300 cycles with a capacity retention of 86.4%, while the pristine one only delivers 154.1 mAh g^−1^ with a capacity retention of 76.9%. The capacity fading for the pristine NCM811 is presumably due to the structural transition and interface side reaction upon cycling,^[^ ^34^ ^]^ and the superior electrochemical properties of the doped samples should be attributed to its more stable structure and interface chemistry.

To further verify the improved structural stability of the modified sample, the different charge–discharge and dQ dV^−1^ studies were performed and are shown in Figure S8, Supporting Information. It can be clearly seen from Figure S8a,b, Supporting Information, that the capacity attenuation (especially for the voltage decay) is effectively mitigated by ZrB_2_ doping and the structure of 0.2ZB‐NCM811 during the cycling can be effectively maintained. dQ dV^−1^ curves further confirm the structural changes during the charging and discharging process (Figure S8c,d, Supporting Information). Similar to other Ni‐rich cathodes, NCM811 and 0.2ZB‐NCM811 generally undergo a series of phase transitions during the charging, such as H1 (hexagonal) ↔ M (monoclinic), M (monoclinic) ↔ H2 (hexagonal), and H2 (hexagonal) ↔ H3 (hexagonal). The final phase transition of H2 ↔ H3 has been proven to be a major reason for capacity attenuation as it is closely related to the lattice distortion and microcrack formation.^[^ ^35^ ^]^ As shown in Figure S8c, Supporting Information, the H2 ↔ H3 oxidation peak for the NCM811 electrode gradually shifts to a higher voltage with cycling. The intensity of the peak also gradually decreases, indicating serious irreversibility of the H2 ↔ H3 phase transition. For comparison, the H2 ↔ H3 oxidation peak for the 0.2ZB‐NCM811 electrode is well maintained even after 100 cycles (Figure S8d, Supporting Information), revealing that the irreversibility of the H2 ↔ H3 transition could be effectively suppressed by ZrB_2_ doping. Besides, a similar phenomenon can be observed when tested at high temperature (Figure S9, Supporting Information). This further confirms the advantages of ZrB_2_ doping for stabilizing the structure of Ni‐rich cathode material.

**Figure 6:**
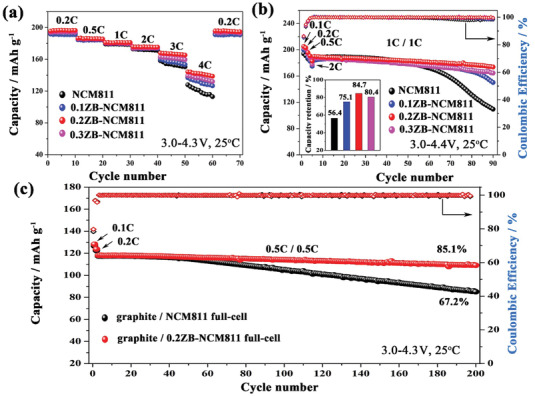
Electrochemical characterization of as‐prepared samples: a) rate capability; b) galvanostatic cycling with the upper cutoff potential of 4.4 V; c) cycling performance at 0.5C rate for full cells (calculated based on the mass of positive and negative active materials).

Rate performances for as‐prepared samples are displayed in **Figure** **6**a. All samples deliver a high specific capacity of ≈192 mAh g^−1^ at a low rate of 0.2C, which accounts to 69.5% of the theoretical capacity of LiNiO_2_. However, the discharge capacity for prepared samples decreases with the continuous increase in the C‐rate, and the 0.2ZB‐NCM811 delivers the highest discharge capacity of 168.1 mAh g^−1^ (9.8 mAh g^−1^ higher than that of the pristine one) at 3C rate. Note that the discharge capacities of all doped samples at 3C and 4C are much higher than that of the pristine one, indicating that rate performance can be improved after ZrB_2_ treatment. The excellent rate performance of the modified sample should be attributed to the higher lithium‐ion diffusion coefficient (*D*_Li_^+^) due to the enlargement of the unit cell (Figure S10, Supporting Information),^[^ ^36^ ^]^ indicating that ZrB_2_ doping can offer a suitable framework to facilitate ion transport kinetic.

The influence of three in one strategy of adding ZrB_2_ on the electrochemical properties was further investigated. We have increased the cutoff voltage from 4.3 to 4.4 V and cycling performances are compared in Figure 6b. Unfortunately, the pristine NCM811 shows fast capacity fading only after 60 cycles. The capacity retention decreases to 56.4% after 90 cycles. It is well known that high cutoff voltage will cause severe interfacial side reactions and phase transitions due to excessive delithiation, resulting in a larger capacity loss for Ni‐rich cathode.^[^ ^37^ ^]^ Unexpectedly, the 0.2ZB‐NCM811 still maintains good cycling stability. It delivers a specific discharge capacity of 173.7 mAh g^−1^ with a capacity retention of 84.7% after 90 cycles. Furthermore, in order to study the commercial application prospects for the modified samples, full cells consisting of graphite anode were assembled. The full cell of graphite/0.2ZB‐NCM811 demonstrates superior cycling stability (Figure 6c). Even after 200 cycles, it could deliver an impressively high discharge capacity of 109.1 mAh g^−1^ with 85.1% capacity retention (calculated based on the mass of positive and negative active materials). However, the pristine sample only shows a capacity retention of 67.2%.

Figure S11a–f, Supporting Information, summarizes the morphologies of the electrodes before and after cycling. Initially, the particles are uniformly distributed on the current collector (Figure S11a,b, Supporting Information). After 100 cycles at 25 °C, microcracks are observed in the pristine electrode (Figure S11c, Supporting Information), and these cracks are aggravated when cycled at 50 °C (Figure S11e, Supporting Information). While the modified particles show better stability (both at 25 and 50 °C) as its morphology remains intact (Figure S11d,f, Supporting Information). The corresponding kinetic behavior and resistance parameters were analyzed by electrochemical impedance spectroscopy (EIS) as shown in Figure S11g–i, Supporting Information. The fitted equivalent circuits are provided in Figure S12, Supporting Information. The Nyquist plots comprise of a sloped line and two semicircles and the interpretation of the sloped line and semicircles have been discussed in previous literature.^[^ ^38^ ^]^ Clearly, after 100 cycles at 25 °C, the impedance values (*R*_sf_ + *R*_ct_) of NCM811 and 0.2ZB‐NCM811 increase to 117.7 and 79.6 Ω from 31.1 and 24.3 Ω, respectively. Furthermore, the (*R*_sf_ + *R*_ct_) value of NCM811 increases significantly and reaches 415.2 Ω when tested at 50 °C, while the 0.2ZB‐NCM811 shows a lower impedance of 262.3 Ω. These results indicate that the ZrB_2_ treatment could greatly reduce the lithium‐ion diffusion resistance and improve the electrochemical stability upon cycling.

**Figure 7:**
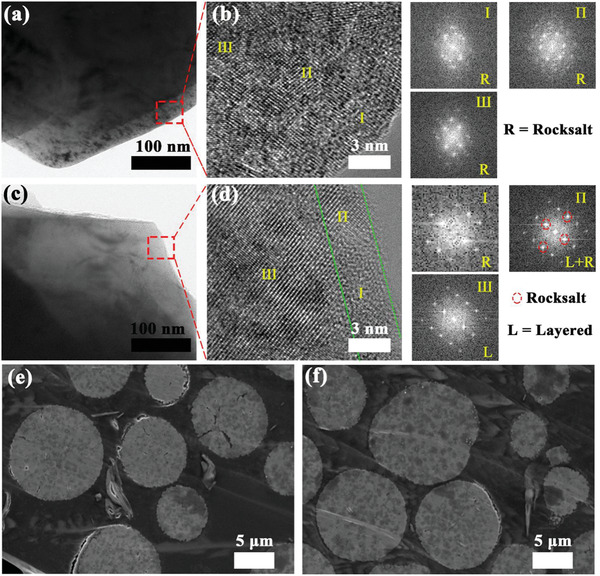
Low‐magnification TEM images of a) NCM811 and c) 0.2ZB‐NCM811 particles after 100 cycles, b,d) the corresponding high‐magnification TEM images, and Fourier filtered transforms of the regions marked by numerals in (b) and (d); cross‐sectional SEM images of e) NCM811 and f) 0.2ZB‐NCM811 particles after 100 charge and discharge cycles at 25 °C.

To further validate the stability of the modified sample, the local atomic composition and interfacial microstructure of NCM811 and 0.2ZB‐NCM811 after 100 cycles were studied in detail. The results are depicted in **Figure** **7**. Apparently, the doped particle exhibits a smooth surface while the pristine one presents a rough surface (Figure 7a,c). A big difference appears in the high‐resolution images. For the pristine sample, the layered structure is almost damaged due to the parasitic reaction with the electrolyte (Figure 7b), and many NiO‐like rock‐salt structures have formed according to the analyzed Fourier transform images.^[^ ^39^ ^]^ The Ni^2+^ ions in rock‐salt phase derived from the reduction of unstable Ni^4+^ ions, which is accompanied by the increase in electrode impedance and oxygen release, resulting in the deterioration of structural stability and the poor thermal stability. Interestingly, the cycled particle of 0.2ZB‐NCM811 only shows ≈3 nm disorder mixed phases, with layered and rock salt phases co‐existing on the surface (Figure 7d; after 100 cycles), and well‐retained lattice fringes in the inner‐regions. This indicates that the irreversible phase transition in the Ni‐rich cathode mainly initiates from the crystal surface, and gradually spreads to the core with cycling.^[^ ^40^ ^]^ In addition, the coating layer is almost damaged or disappears after 100 cycles (Figure 7d), which could be attributed to the side reactions between the coatings and the electrolyte. This demonstrates that the strategy present in our work is beneficial for improving the interfacial stability of the Ni‐rich cathode. To in‐depth investigate the bulk structural evolution for the secondary particle in as‐prepared layered materials after 100 cycles, the cross‐sectional SEM images are given in Figure 7e,f and Figure S13, Supporting Information. Numerous intragranular cracks emanating from the high level of intrinsic strain are observed in the NCM811 particle (Figure 7e; Figure S13c, Supporting Information). This strain could cause the entire particles to crack and open up pathways for the electrolyte to penetrate the interior of active particle, resulting in structural damage.^[^ ^41^ ^]^ In contrast, only some microcracks emanating from the core are observed for 0.2ZB‐NCM811 particle (Figure 7f; Figure S13d, Supporting Information), which reveals the positive effect of ZrB_2_ doping for intergranular crack suppression during the long‐term cycling. Hence, we believe that the capacity fading in Ni‐rich cathode caused by intragranular cracks and structural phase transition can be greatly mitigated through ZrB_2_ doping.

**Figure 8:**
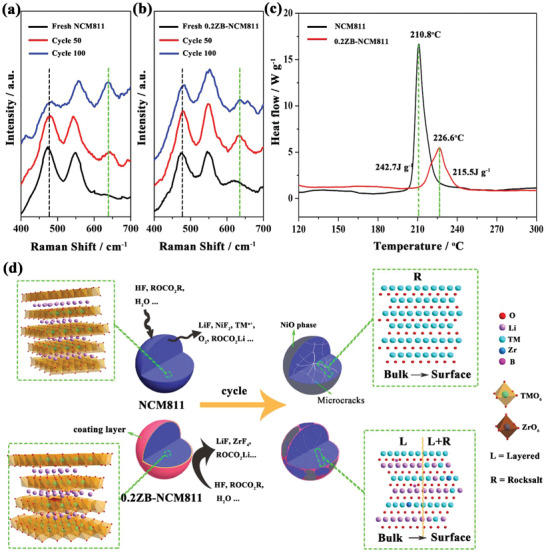
Raman spectra of a) NCM811 and b) 0.2ZB‐NCM811 electrodes before and after various electrochemical cycles between 3.0 and 4.3 V; c) DSC profiles of NCM811 and 0.2ZB‐NCM811 cathode materials at the charged state of 4.3 V; d) schematic illustration of the effect of ZrB_2_ doping on the NCM811 cathode's mechanical stability during cycling.

To examine the improved structural stability of 0.2ZB‐NCM811 cathode, the ex situ Raman spectroscopies were obtained before and after various electrochemical cycles between 3.0 and 4.3 V in **Figure** **8**a,b. It is clear that the layered structure of the pristine sample was deteriorated after 100 cycles as proven by the almost disappeared band at ≈465 cm^−1^ (belongs to the characteristic diffraction peak of the layered structure, marked with black dash line) and the strengthened band at ≈630 cm^−1^ (belongs to the characteristic diffraction peak of the spinel‐like structure, marked with green dash line; Figure 8a).^[^ ^11e^ ^]^ However, for the modified sample, 0.2ZB‐NCM811, the band at ≈465 cm^−1^ is clearly detected even after 100 cycles (Figure 8b), indicating that the layered structural stability can be improved after ZrB_2_ doping. Furthermore, differential scanning calorimetry (DSC) profiles confirm that the thermal stability is obviously enhanced for the doped sample (Figure 8c), as the exothermic peak shifts from 210.8 to 226.6 °C and the heat generation decreases from 242.7 to 215.5 J g^−1^ after ZrB_2_ treatment.

For better understanding the critical role of three in one strategy in this work, a comparative scheme is illustrated in Figure 8d. Generally, for bare NCM811 electrode, there are severe interfacial side reactions originating from electrolyte decomposition and HF attack, which result in TM dissolution and oxygen release, subsequently, cause the rock‐salt phase formation on the surface. Meanwhile, the lattice distortion and anisotropic volume changes derived from the different changes in cell parameters will result in localized stress concentrations of primary particles and subsequent microcracking of secondary particles, thereby accelerate the bulk structural degradation of the NCM811 cathode. The good news is that the three in one strategy of ZrB_2_ doping has well addressed such issues. The Zr and B co‐doping exhibits great application potential compared with the previously reported Ni‐rich cathode materials (Table S3, Supporting Information).^[^ ^13^, ^14^, ^17^, ^42^ ^]^ The formed lithium zirconium oxide coating layer after the modification of Ni‐rich cathode material with ZrB_2_ can not only protect the surface layered structure but also prevent the TMs dissolution from the electrolyte. Furthermore, the bulk doping of Zr and B can alleviate the lattice distortion and relieve the intrinsic internal strain, resulting in better mechanical integrity of the secondary particles in the 0.2ZB‐NCM811 cathode during lithiation/delithiation process. More importantly, it can avoid the electrolyte infiltration into the particle interior and thus prevent the internal phase transition in particles as well as reduce the loss of active lithium. Based on the above analysis, in this paper, the bulk doping maybe more important than the surface modification because of the intrinsic structural stability can be improved after Zr and B doping.

## 3 Conclusions

In this study, Zr doping, B doping, and interfacial coating were achieved for stable LiNi_0.8_Co_0.1_Mn_0.1_O_2_ cathode by a one‐step strategy using ZrB_2_ dopant. Owing to this three in one strategy, the treated cathodes exhibited much superior electrochemical properties in comparison with the pristine sample. Typically, the 0.2 mol% ZrB_2_‐LiNi_0.8_Co_0.1_Mn_0.1_O_2_ delivered a discharge capacity of 183.1 mAh g^−1^ (at 1C and 3.0–4.3 V) after 100 cycles at 50 °C with an outstanding capacity retention of 88.1%. Even at a higher cut‐off voltage of 4.4 V, it displayed exceptionally high cycling stability (>90 cycles) with 84.7% capacity retention which was much higher than that of the pristine sample. First‐principles calculations and experimental analysis revealed that the markedly improved cycling performance and thermal stability of doped samples are due to the a) higher formation energy of oxygen vacancies, b) higher exchange energy of Li/Ni exchange, c) lower lattice distortion, d) more stable O^2−^ anion framework, e) enhanced reversibility of the H2 ↔ H3 phase transition, and f) suppression of the intergranular cracks during the cycling. This three in one strategy could provide rational guidance to design high‐performance Ni‐rich cathode materials, thereby promoting the development of high‐energy‐density LIBs.

## 4 Experimental Section

##### Materials Preparation

Ni_0.8_Co_0.1_Mn_0.1_(OH)_2_ precursor was synthesized via a co‐precipitation method which had been reported in the previous work.^[^ ^43^ ^]^ The Ni_0.8_Co_0.1_Mn_0.1_(OH)_2_ precursor, ZrB_2_ nanoparticles (provided by Beijing Deke Daojin Science and Technology Co., Ltd.), and LiOH∙H_2_O with Li:TM:ZrB_2_ ratio of 1.05:1:0.001 were thoroughly mixed by ball milling. Excess lithium (0.05 mol%) was added to compensate the lithium loss when sintering at high temperature. This mixture was heated to 650 °C for 5 h with a ramp rate of 1.5 °C min^−1^ in the O_2_ atmosphere and then sintered at 800 °C for 10 h with a ramp rate of 0.75 °C min^−1^ to obtain the modified cathode of 0.1% ZrB_2_‐LiNi_0.8_Co_0.1_Mn_0.1_O_2_ (denoted as 0.1ZB‐NCM811). The samples of NCM811, 0.2ZB‐NCM811, and 0.3ZB‐NCM811 were obtained with the same procedure by using different proportions of ZrB_2_.

##### Materials Characterization

Crystalline structures of the as‐synthesized powder samples were determined using a Rigaku Ultima III diffractometer with a Cu‐Ka radiation source (*λ* = 0.15406 nm), and the Bragg angle was tested over a range of 10–80° at a scan rate of 2° min^−1^. The lattice parameters of the samples were analyzed by the FULLPROF Rietveld program. In situ XRD experiments were performed using the Bruker D8 Advance diffractometer (Cu K*α* radiation (*λ* = 0.154 nm), 15° ≤ 2*θ* ≤ 80°). The self‐constructed cells were tested galvanostatically between 3.0 and 4.3 V at a rate of 20 mA g^−1^. The morphology, microstructure, and elemental distribution of the powders were characterized by focused ion beam scanning electron microscopy (Helios Nanolab G3), and the electrode morphologies before and after cycling were observed by SEM (FEI Verios 460). The surface chemical properties of the prepared samples were determined by XPS (Thermo ESCALAB 250XI). The particle morphology and structure of the as‐prepared samples before and after 100 cycles were studied by HR‐TEM (Jeol 2100F). The cycled and charged electrodes were obtained after disassembling the cycled batteries inside the Ar‐filled glovebox and were repeatedly washed by DMC to remove residual electrolyte. The cycled and charged active materials were recovered by sonicating the cycled electrodes in *N*‐methylpyrrolidinone (NMP) for 10 min followed by filtering, washing, and drying at room temperature. The thermal stability of the charged samples (4.3 V) was determined after by DSC at an operating step temperature of 5 °C min^−1^ (TAQ2000).

##### Electrochemical Characterization

To analyze the electrochemical performances of the prepared samples, the positive electrodes were fabricated by mixing the active materials, super P (aladdin) and polyvinylidene fluoride in a weight ratio of 92.5:4.5:3 in NMP (aladdin) solution, which was more close to the practical requirement. The obtained slurry was uniformly cast on Al foil with an active material loading level of ≈5 mg cm^−2^. The electrodes were dried in an oven at 120 °C for 12 h and roll pressed. Half cells were assembled in an Ar‐filled glovebox using a 2016 coin‐type cell with the commercial lithium disk as the anode, while the full cells were assembled in a 2025 coin‐type cell with the commercial graphite (provided by BTR New Energy Material Ltd, ≈2.9 mg cm^−2^) as the anode. 1.0 mol L^−1^ LiPF_6_ solution dissolved in ethylene carbonate‐ethyl methyl carbonate mixture (EC: EMC = 3:7 by volume) with 2 wt% vinylene carbonate was used as the electrolyte. The electrochemical tests were performed at different current rates (1C = 200 mA g^−1^) using LAND test system (CT2001A) between 3.0 and 4.3 V (vs Li/Li^+^) at 25 and 50 °C, respectively. The EIS studies were done at an amplitude of 5 mV in the frequency range of 0.01 Hz to 100 kHz using PARSTAT 4000A electrochemical workstation. The Li^+^ diffusion properties (*D*_Li_^+^) of the samples were analyzed by galvanostatic intermittent titration technique.

## Conflict of Interest

The authors declare no conflict of interest.

## Supporting information

Supporting Information
