# Supplementary material for: A Three in One Strategy to Achieve Zirconium Doping, Boron Doping, and Interfacial Coating for Stable LiNi0.8Co0.1Mn0.1O2 Cathode
Source: Adv Sci (Weinh). 2020 Nov 27;8(2):2001809. doi: 10.1002/advs.202001809 (PMC7816706; doi:10.1002/advs.202001809)
Supplement: Supplementary file 1 — Supporting Information [file ADVS-8-2001809-s001.pdf]

© 2020 Wiley-VCH GmbH

ADVANCED SCIENCE
Open Access

Supporting Information

for *Adv. Sci.*, DOI: 10.1002/advs.202001809

A Three in One Strategy to Achieve Zirconium Doping, Boron Doping and Interfacial Coating for Stable $LiNi_{0.8}Co_{0.1}Mn_{0.1}O_2$ Cathode

Ze Feng, Ranjusha Rajagopalan, Shan Zhang, Dan Sun, Yougen Tang, Yu Ren, and Haiyan Wang**

© 2020 Wiley-VCH GmbH

Supporting Information

A three in one strategy to achieve zirconium doping, boron doping and interfacial coating for stable $LiNi_{0.8}Co_{0.1}Mn_{0.1}O_2$ cathode

*Ze Feng[a], Ranjusha Rajagopalan[a], Shan Zhang[a], Dan Sun[a], Yougen Tang[a, *], Yu Ren[b], Haiyan Wang[a, *]*

Computational method:

Density of functional theory (DFT) calculations were implemented using the projector-augmented wave (PAW) method with the Vienna ab initio simulation package (VASP). The generalized gradient approximation with Perdew–Burke–Ernzerhof (GGA-PBE) was used as the exchange-correlation function. The spin polarized was chosen during the whole calculations. The kinetic energy cutoff and the maximum SCF cycles were set to 340 eV and 3000. To improve calculation accuracy, the Hubbard-type U correction was taken into account on the transition metal and the Hubbard U parameters of Ni, Mn, Co and Zr were chosen to be 6.4, 4.9, 4.5 and 2.0 eV, respectively. The gamma centered Monkhorst–Pack scheme of k-point generation is applied to grids of 3 × 3 × 1. During the structural optimization, the atomic positions of all atoms were fully relaxed until the Hellmann–Feynman force acting on each atom was <0.01 eV $Å^{-1}$.

To study the doping process of the different incorporated elements, the model surfaces of $Li_{36}Ni_{21}Co_3Mn_3O_{63}$ (001) plane (3 × 3 supercell of 126 atoms) with single Zr or B and dual Zr-B on the topmost surface have been constructed (Figure S5). The Zr and B atoms were added into the bulk of $Li_{36}Ni_{21}Co_3Mn_3O_{63}$ (001) slab (Figure S5), and the migration energy (ΔE) was calculated according to the follow equations.

$$\Delta E(Zr) = E(Samp^{Zr\text{-}doped}) - E(Samp) + \mu(Ni) \qquad (1)$$

$$\Delta E(B) = E(Samp^{B\text{-}doped}) - E(Samp) \qquad (2)$$

Where E($Samp^{Zr\text{-}doped}$) and E($Samp^{B\text{-}doped}$) denote the total energy with the incorporation of Zr and B. E(Samp) denotes the total energy when Zr, B, and dual Zr-B adsorbed on the surface.

To better understand the effect of ZrB_2 doping on the bulk structure, the model with a 3 × 3 × 1 supercell of 108 atoms of α-$NaFeO_2$ type ($Li_{27}Ni_{21}Co_3Mn_3O_{54}$, space group R-3m) unit cell have been built to optimize the structures and calculate the energies of bare and doped samples (Figure S6). The concentration of ~ 1% Zr was used to replace the TM ions of Ni site[1], while ~ 1% B was used to dope the tetrahedral interstices site of the oxygen layer (ZB-$Li_{27}Ni_{20}Co_3Mn_3O_{54}$)[2]. The formation energy of oxygen vacancies (V_O) and the exchange energy of Li/Ni exchange were defined based on the following equations[3], respectively.

$$\Delta E(Vo) = E(Samp^{Vo}) + 1/2\,E(O_2) - E(Samp) \qquad (3)$$

$$\Delta E(Li/Ni) = E(Samp^{Li/Ni}) - E(Samp) \qquad (4)$$

Wherein, $\Delta E(V_O)$ and $\Delta E(Li_/Ni)$ represent the formation energy of oxygen vacancies and the exchange energy of Li/Ni exchange. The E(Samp) and E($Samp^{Vo}$) represent the total energies of the corresponding samples before and after losing one oxygen atom, and The E($Samp^{Li/Ni}$) represents the total energy of the corresponding sample after Li/Ni exchange.

In order to further investigate the effect of Zr accumulation on the oxygen. The model surface of bare $Li_{24}Ni_{20}Co_2Mn_2O_{48}$ (104) plane and modified $Li_{24}Ni_{16}Co_2Mn_2Zr_4O_{48}$ (2 × 3 supercell of 96 atoms) plane with Zr accumulates at the surface have been constructed and shown in Figure S7. Compared with the bare sample, the Zr is used to replace the transition metal ions

on the surface. The formation energy of surficial oxygen vacancies (V_O) is calculated according to the equation 3.

GITT measurement:

For better understand the electrochemical properties of the bare and modified samples, Galvanostatic intermittent titration technique (GITT) is conducted to calculate the Li^+ diffusion coefficient ($D_{Li}{}^+$) by using 0.1 C charging process and 40 min interval in 3.0–4.3 V. Before the test, the batteries firstly underwent a charge-discharge circle of activation at 0.1C. The values of $D_{Li}{}^+$ can be calculated according to equation 5.

$$D_{Li^+} = \frac{4}{\pi}\left(\frac{m_B V_m}{M_B S}\right)^2\left(\frac{\Delta E_S}{\Delta E_\tau}\right)^2 \quad (5)$$

Where m_B represents the active material mass, V_m ($cm^3\ mol^{-1}$) refers to the molar volume, M_B is the molar mass, and S (cm^2) is the total contact area. ΔE_S is the steadystate voltage change, due to the current pulse and ΔE_τ is the voltage change during the constant current pulse, eliminating the iR drop.

Figure. S1

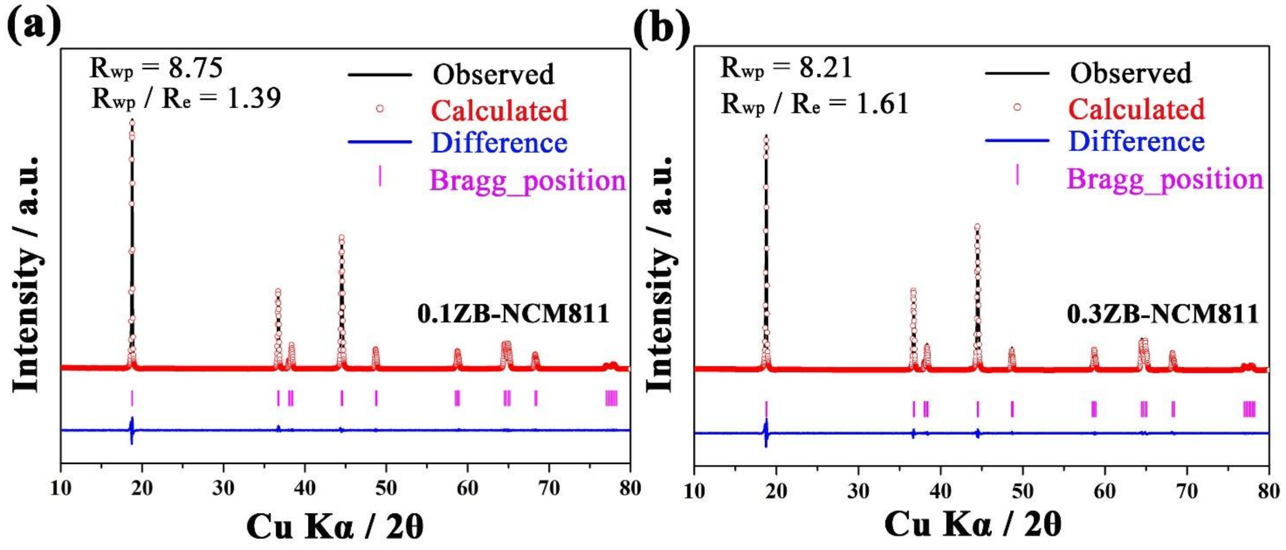

Figure. S1 Rietveld refinement results of 0.1ZB-NCM811 and 0.3ZB-NCM811.

Figure. S2

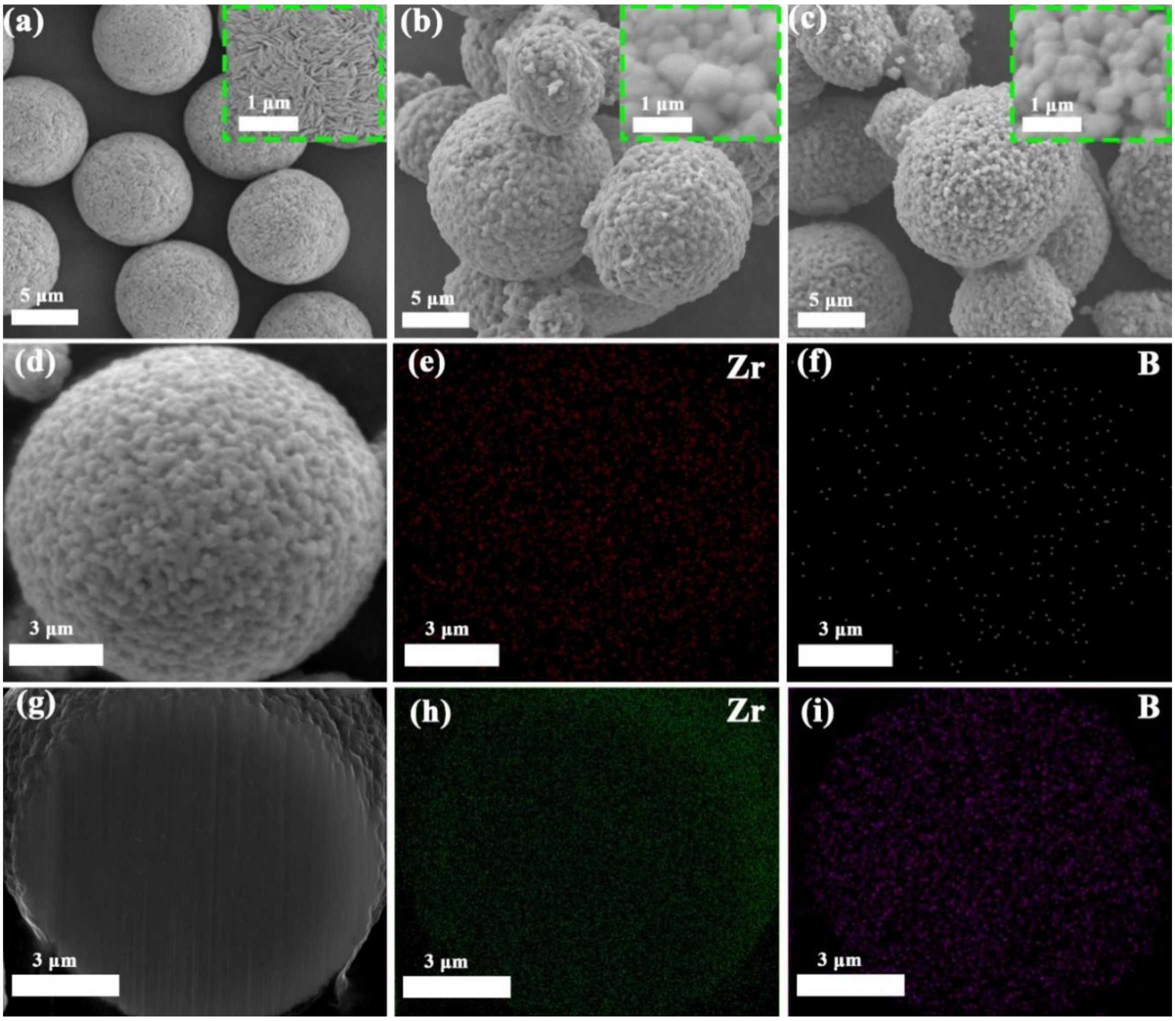

Figure. S2 SEM images of as-prepared (a) $Ni_{0.8}Co_{0.1}Mn_{0.1}(OH)_2$, (b) NCM811, (c) and (d) 0.2ZB-NCM811, (e) - (f) Elemental mapping images of 0.2ZB-NCM811; (g) Cross-sectional SEM images of 0.2ZB-NCM811, (h) - (i) are the elemental mapping results of Zr and B.

Figure. S3

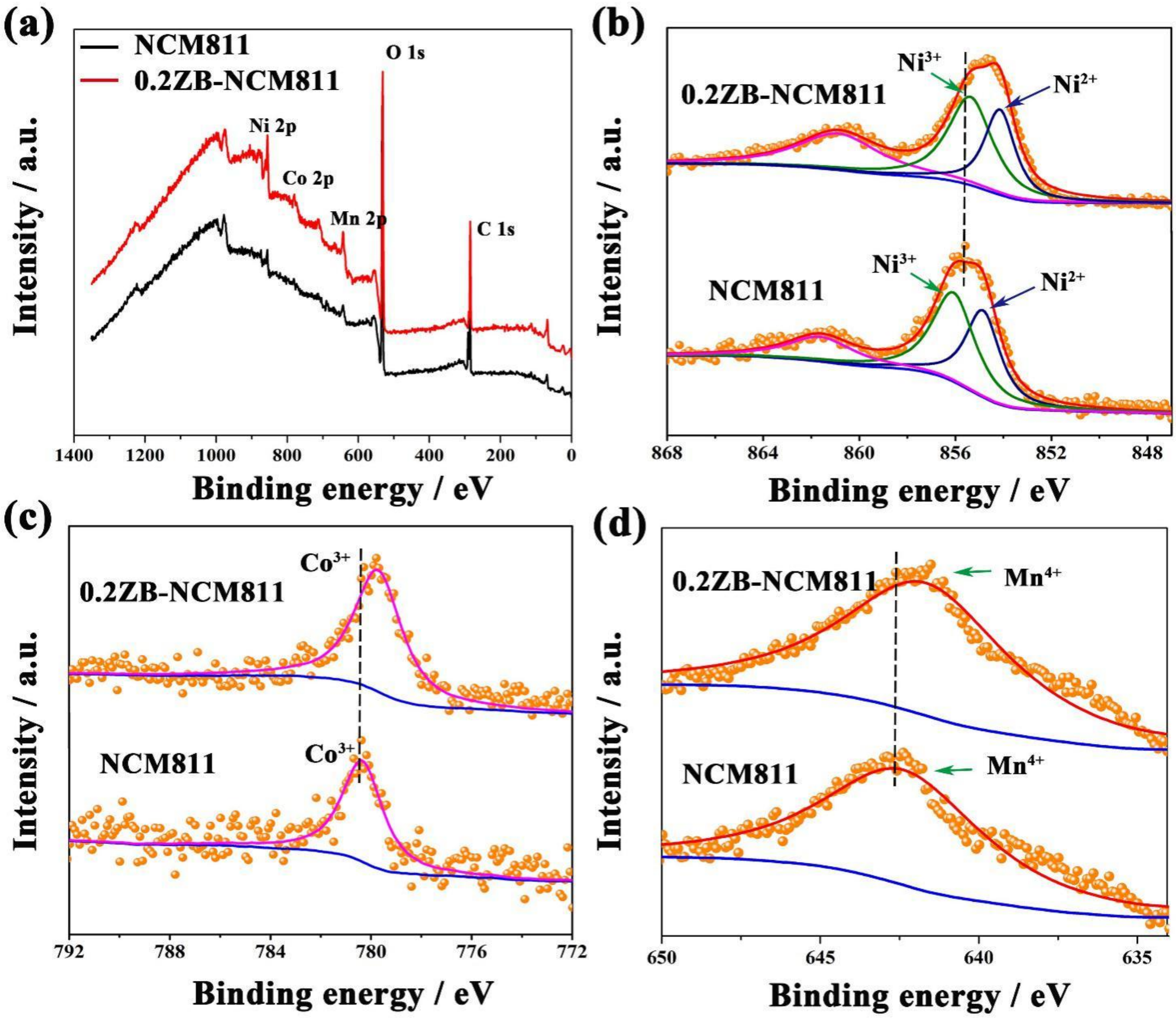

Figure. S3 (a) XPS survey spectra of NCM811 and 0.2ZB-NCM811; (b) - (d) XPS spectra of Ni, Co and Mn of NCM811 and 0.2ZB-NCM811.

Figure. S4

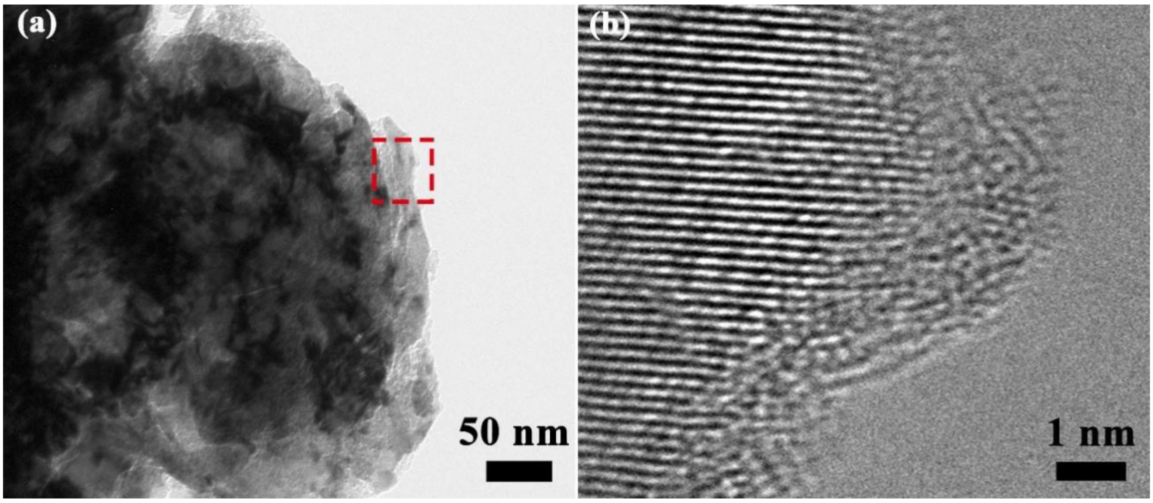

Figure. S4 (a) TEM images of 0.2ZB-NCM811; (b) HR-TEM images of 0.2ZB-NCM811;

Figure. S5

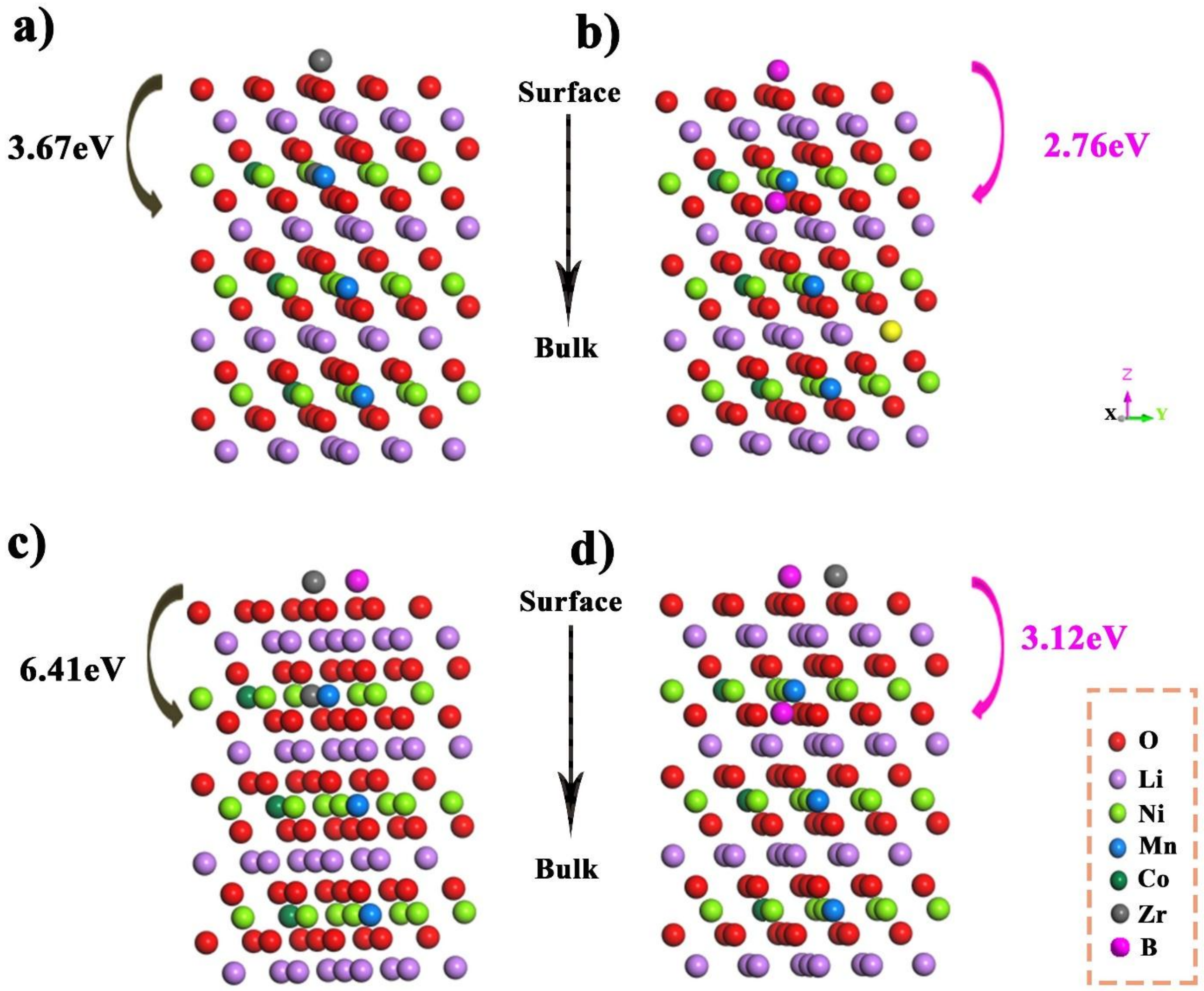

Figure S5. Side view of the structures (a) Zr and (b) B incorporated $Li_{36}Ni_{21}Co_3Mn_3O_{63}$ (001) plane; (c) single Zr and (d) single B incorporated $Li_{36}Ni_{21}Co_3Mn_3O_{63}$ (001) plane when the Zr and B coexist on the surface, respectively.

Figure. S6

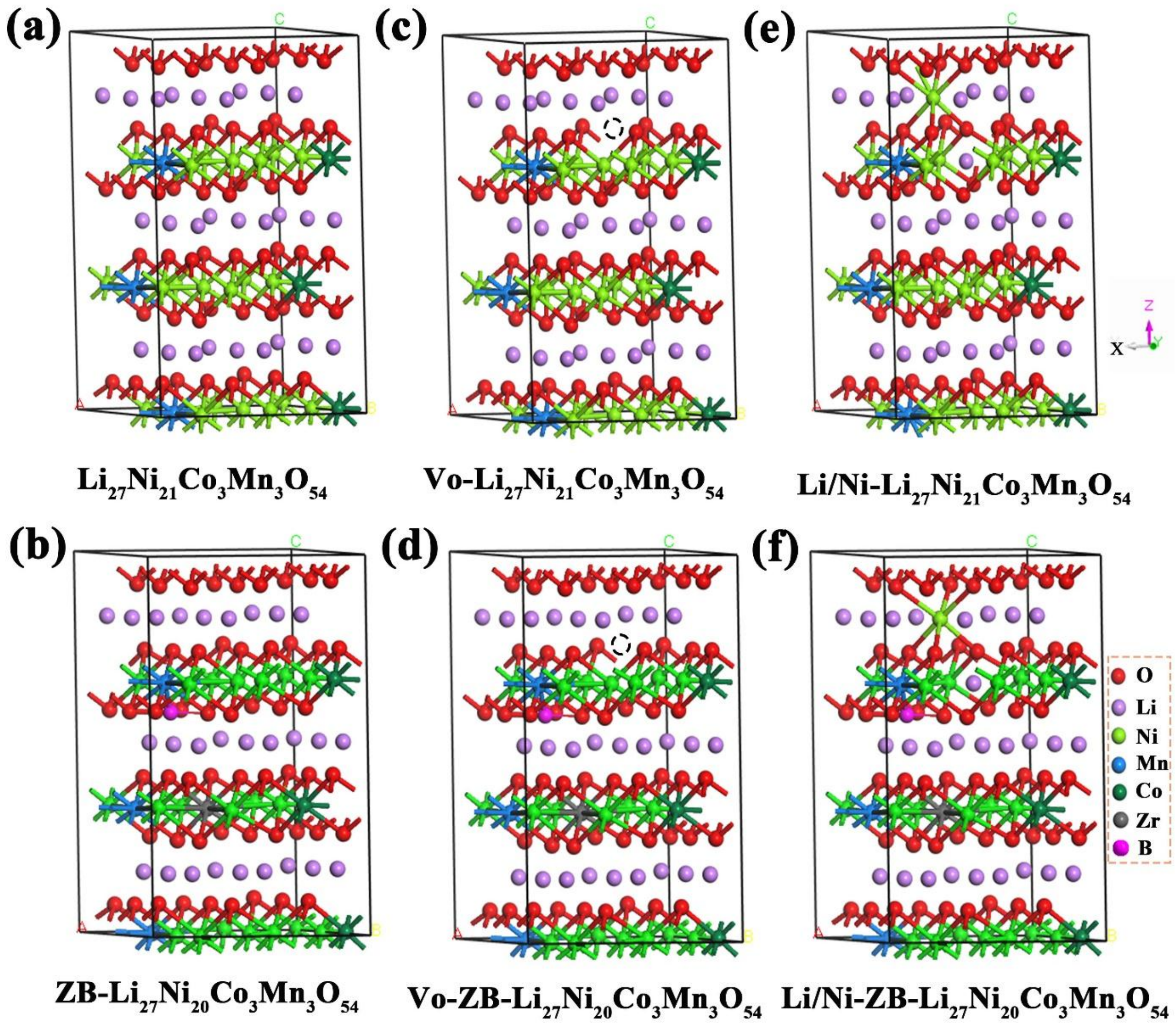

Figure. S6 Atomic configurations for the structure of (a) $Li_{27}Ni_{21}Co_3Mn_3O_{54}$ and (b) ZB-$Li_{27}Ni_{20}Co_3Mn_3O_{54}$; (c) - (f) the corresponding configuration of oxygen vacancy (Vo) and Li/Ni exchange.

Figure. S7

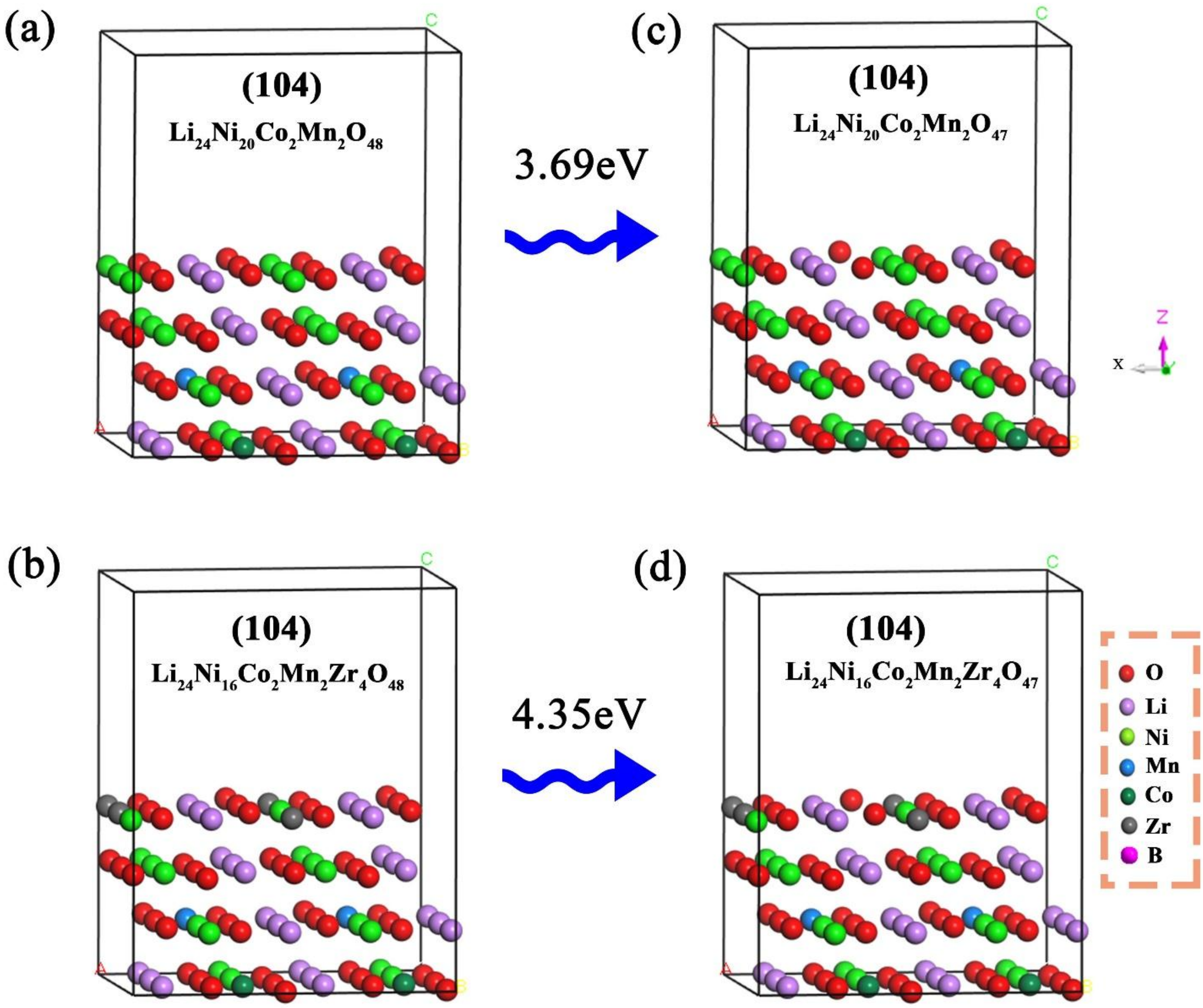

Figure. S7 Structural models for the first-principles calculations to investigate the effects of Zr concentrated on the surface. a) $Li_{24}Ni_{20}Co_2Mn_2O_{48}$ surface constructed along the (104) direction, along with a vacuum slab of 8 Å. b) $Li_{24}Ni_{16}Co_2Mn_2Zr_4O_{48}$ surface after the surface doping of four Zr atoms based on (a), (c) and (d) are the corresponding oxygen vacancy models.

Figure. S8

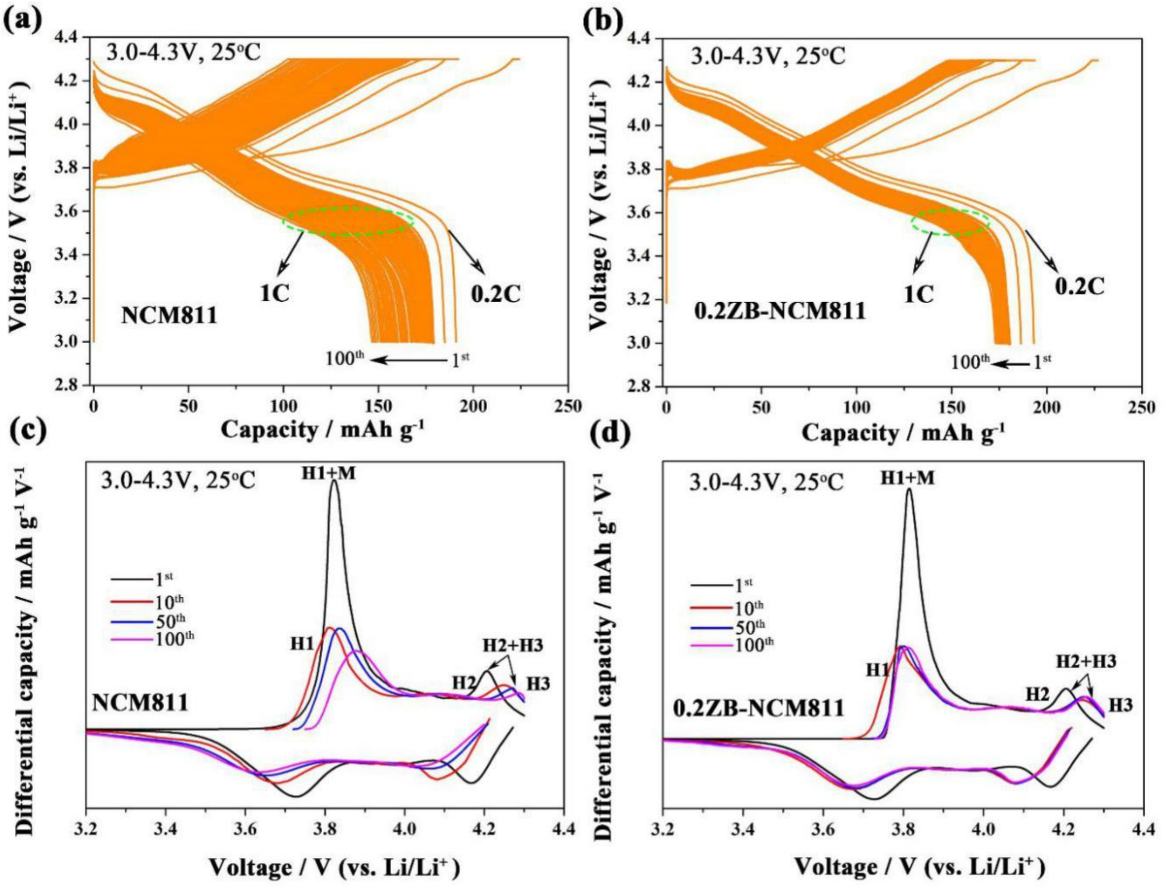

Figure. S8 The charge–discharge profiles of (a) NCM811 and (b) 0.2ZB-NCM811 from 1st to 100th cycles at 25°C; dQ dV^{-1} profiles as a function of the number of cycles for (c) NCM811 and (d) 0.2ZB-NCM811.

Figure. S9

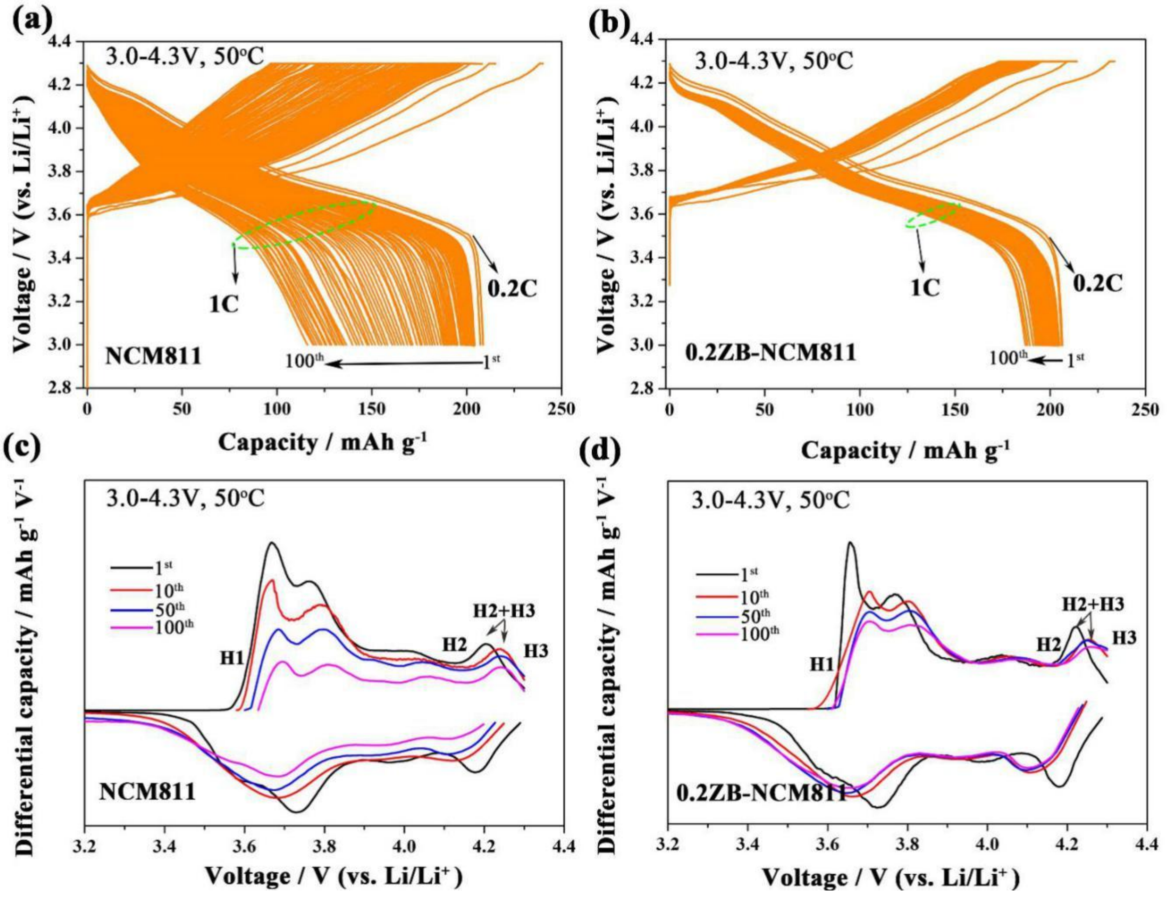

Figure. S9 The charge–discharge profiles of (a) NCM811 and (b) 0.2ZB-NCM811 from 1st to 100th cycles at 50°C; dQ dV^{-1} profiles as a function of the number of cycles for (c) NCM811 and (d) 0.2ZB-NCM811.

Figure. S10

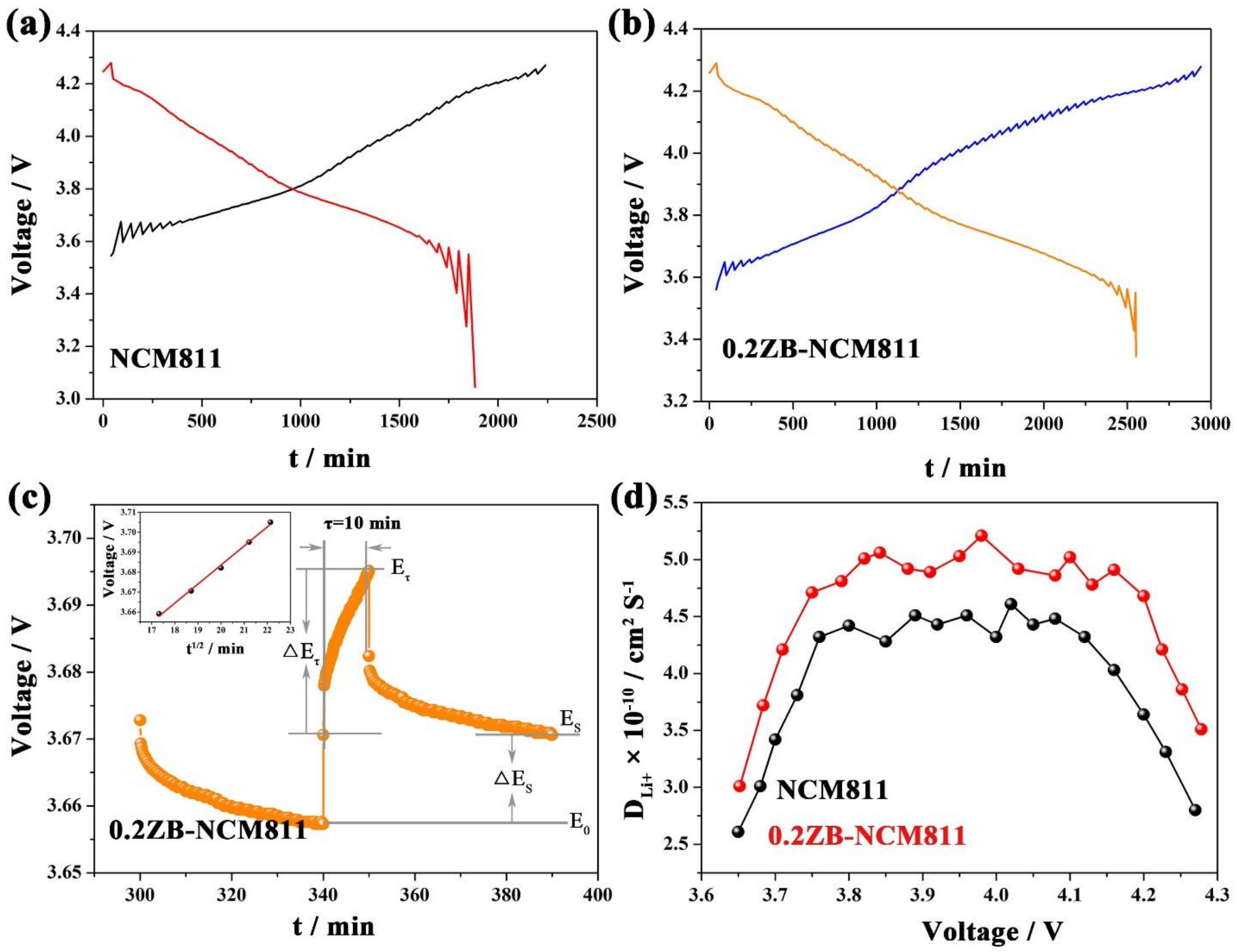

Figure. S10 GITT curves and the corresponding t vs V profiles of (a) NCM811, (b) 0.2ZB-NCM811 cathodes, (c) single titration of 0.2ZB-NCM811, and (d) calculated values of the D_{Li}^{+} for the NCM811 and 0.2ZB-NCM811.

Figure. S11

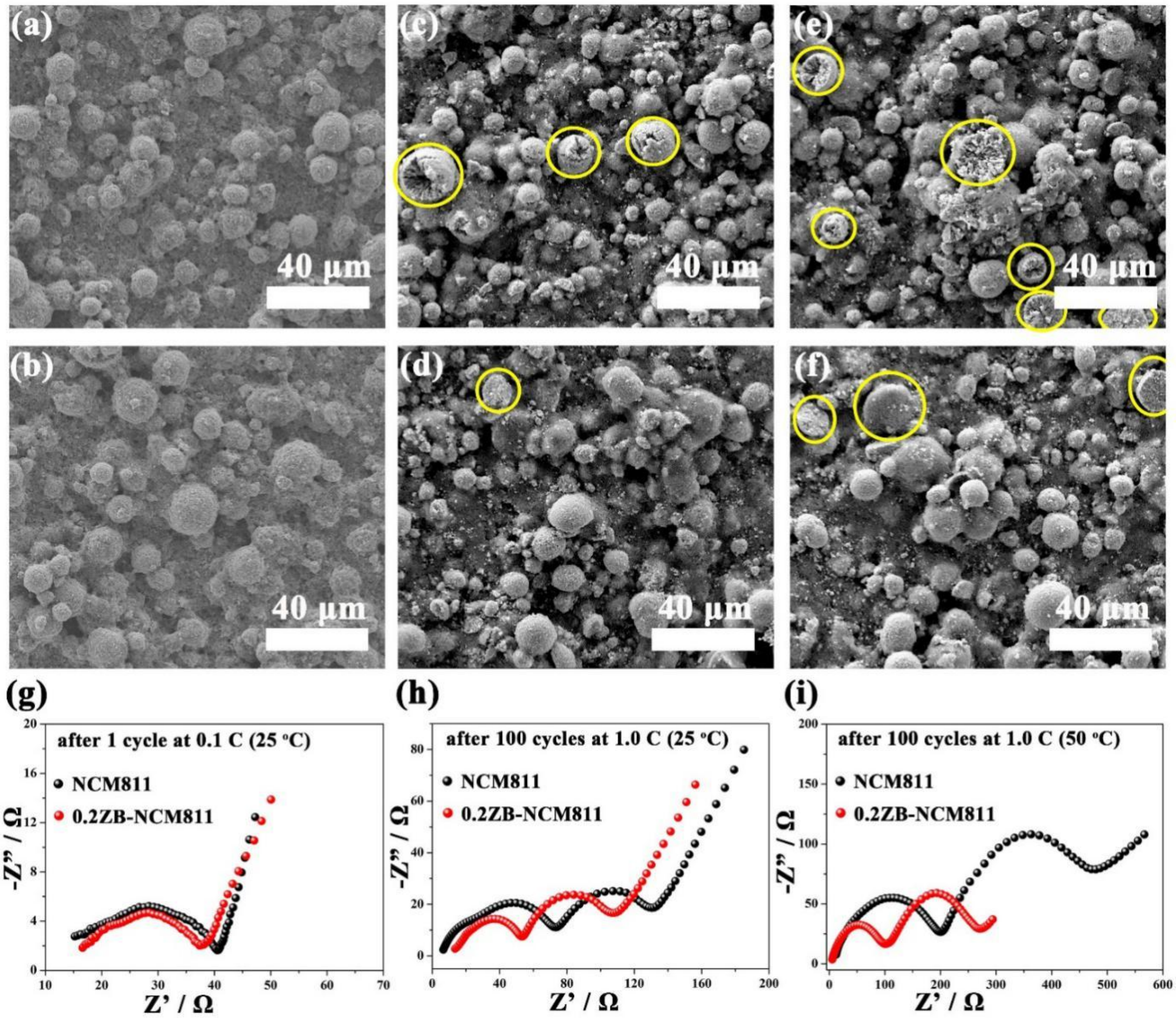

Figure. S11 SEM images of fresh and cycled electrodes: (a) and (b) fresh NCM811 and 0.2ZB-NCM811, (c) and (d) NCM811 and 0.2ZB-NCM811 after 100 cycles at 25°C, (e) and (f) NCM811 and 0.2ZB-NCM811 after 100 cycles at 50°C; The Nyquist plots of the NCM811 and 0.2ZB-NCM811 electrodes: (g) after 1 cycle at 25°C; (h) after 100 cycles at 25°C; and (i) after 100 cycles at 50°C.

Figure. S12

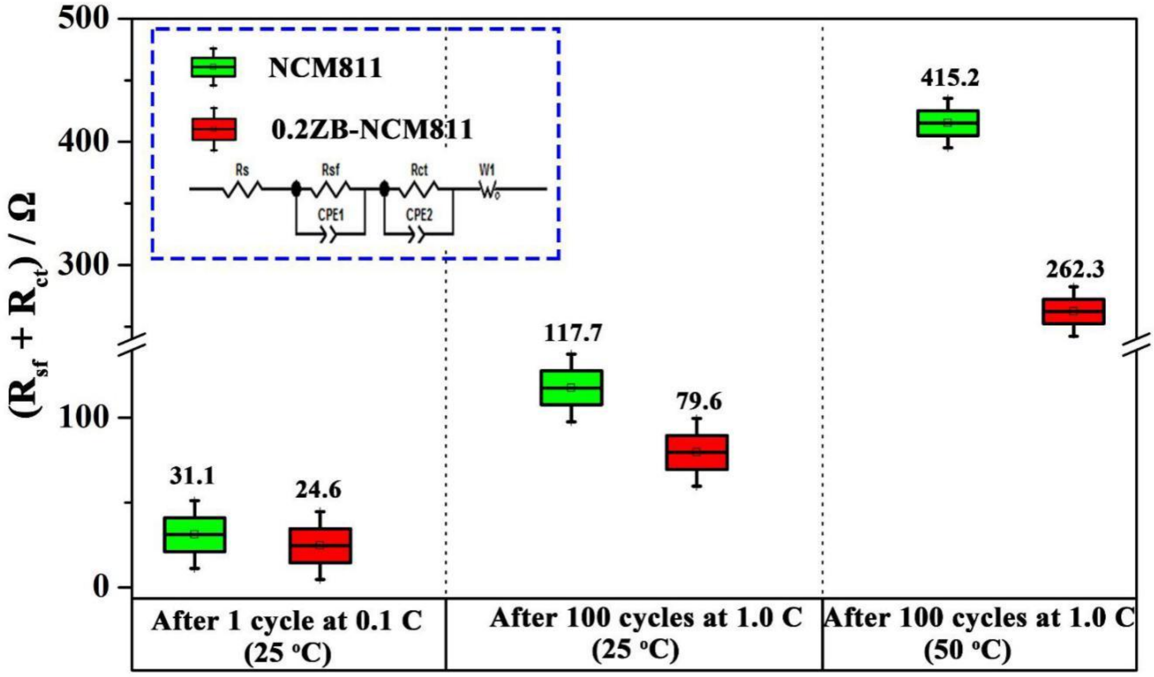

Figure. S12 Resistance values obtained from equivalent circuit by fitting the experimental data for the NCM811 and 0.2ZB-NCM811 electrodes.

Figure. S13

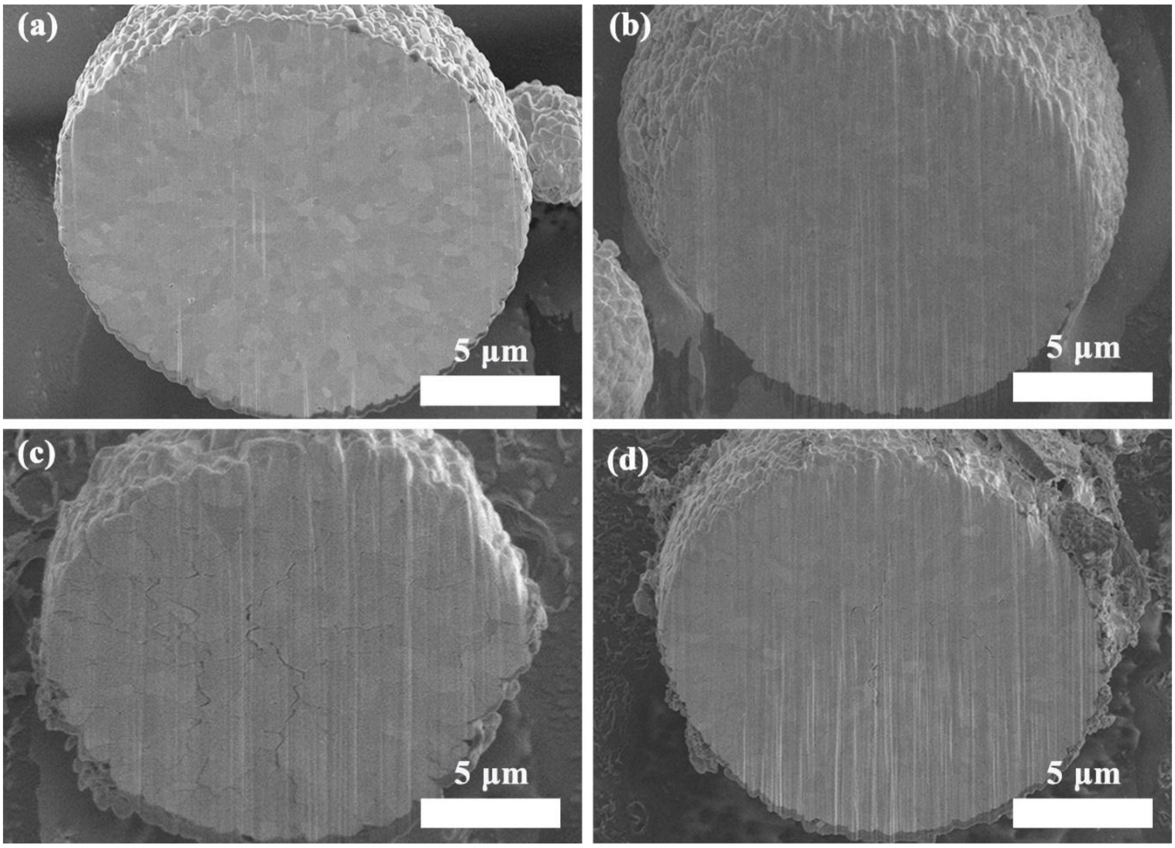

Figure. S13 Cross-sectional SEM images of fresh NCM811 (a) and 0.2ZB-NCM811 particles (b); (e) NCM811 and (f) 0.2ZB-NCM811 particles after 100 charge and discharge cycles at 25°C.

Table S1

Table S1. Rietveld refinement data of XRD pattern for all the materials.

Samples	Lattice parameters				I_{003}/I_{104}	Ni^{2+} in Li^{+} layer (%)	R_{wp}	R_{wp}/R_e
	a (Å)	c (Å)	c/a	V (Å^3)				
NCM811	2.8701	14.1966	4.9463	101.276	1.5249	2.07	9.41	1.51
0.1ZB-NCM811	2.8708	14.1982	4.9457	101.352	1.6121	1.93	8.75	1.39
0.2ZB-NCM811	2.8721	14.2029	4.9451	101.469	1.7181	1.58	7.8	1.53
0.3ZB-NCM811	2.8732	14.2088	4.9453	102.011	1.6987	1.71	8.21	1.61

Table S2

Table S2. The calculated energies for the different structural models.

Models	Samples	E(Samp) eV	E($Samp^{Zr\text{-}doped}$) eV	ΔE(Zr) eV
Doping Process, Figure S5	Zr-$Li_{36}Ni_{21}Co_3Mn_3O_{63}$	-69200.01	-67847.89	3.67
	Zr-$Li_{36}Ni_{21}Co_3Mn_3O_{63}$@B	-69285.62	-67930.76	6.41
		E(Samp)	E($Samp^{B\text{-}doped}$)	ΔE(B)
	B-$Li_{36}Ni_{21}Co_3Mn_3O_{63}$	-67998.62	-67841.13	2.76
	B-$Li_{36}Ni_{21}Co_3Mn_3O_{63}$@Zr	-69285.62	-69282.5	3.12
Bulk Calculation		E(Samp)	E($Samp^{Vo}$)	ΔE(Vo)
	$Li_{27}Ni_{21}Co_3Mn_3O_{54}$	-62269.93	-61833.81	1.78
	ZB-$Li_{27}Ni_{21}Co_3Mn_3O_{54}$	-62286.75	-61850.03	2.38
		E(Samp)	E($Samp^{Li/Ni}$)	ΔE(Li/Ni)
	$Li_{27}Ni_{21}Co_3Mn_3O_{54}$	-62269.93	-62270.07	-0.14
	ZB-$Li_{27}Ni_{21}Co_3Mn_3O_{54}$	-62286.75	-62286.64	0.11
Surface Calculation		E(Samp)	E($Samp^{Vo}$)	ΔE(Vo)
	$Li_{24}Ni_{20}Co_2Mn_2O_{48}$	-56026.68	-55588.64	3.69
	$Li_{24}Ni_{16}Co_2Mn_2Zr_4O_{48}$	-55757.87	-55319.18	4.35

Where the Zr-$Li_{36}Ni_{21}Co_3Mn_3O_{63}$@B represents B atom is on the surface while Zr has doped in the bulk.

Table S3

Table S3. Comparison of electrochemical data of reported Ni-rich electrodes with this work.

Materials	Tested conditions	Initial discharge capacity (mAh g^{-1})	High-rate capacity (mAh g^{-1})	Capacity retention	References
1 mol% Al_2O_3 doped NCM811	2.7-4.3 V, 25 ^oC	~ 180 (0.1 C)	-	78.92% (1.0 C, 200 cycles)	[4]
	2.7-4.3V, 55 ^oC	~185 (0.1 C)	-	75.2% (1.0 C, 100 cycles)	
1 mol% Mo doped NCM811	2.75-4.3 V, 25 ^oC	184.1 (1.0 C)	~ 170 (2.0 C)	92.4% (1.0 C, 100 cycles)	[5]
	2.75-4.5 V, 25 ^oC	194.4 (1.0 C)	-	85.2% (1.0 C, 100 cycles)	
1 mol% Zr doped NCM811	2.8-4.3 V, 25 ^oC	192 (0.1 C)	149 (2.0 C)	84.2% (0.2 C, 60 cycles)	[1a]
	-	-	-	-	
3 mol% Ga doped NCM811	2.8-4.3 V, 25 ^oC	~185 (1.0 C)	180 (2.0 C)	91.2% (1.0 C, 100 cycles)	[6]
	-	-	-	-	

1 mol% B_2O_3 doped NCM90	2.8-4.3 V, 30 °C	~230 (0.1 C)	190 (2.0 C)	91% (0.5 C, 100 cycles)	[7]
1 mol% B_2O_3 doped NCM90	2.8-4.3 V, 55 °C	~235 (0.1 C)	-	91% (0.5 C, 100 cycles)	[7]
1 mol% Te doped NCA	2.7-4.3 V, 30 °C	~216 (0.1 C)	-	89.3% (1.0 C, 100 cycles)	[8]
1 mol% Te doped NCA	-	-	-	-	[8]
0.2 mol% ZB_2 doped NCM811	3.0-4.3 V, 25 °C	~192 (0.2 C)	175 (2.0 C)	89.9% (1.0 C, 100 cycles)	This work
0.2 mol% ZB_2 doped NCM811	3.0-4.3 V, 50 °C	~205 (0.2 C)	-	88.1% (1.0 C, 100 cycles)	This work

References

[1] a) S. Gao, X. Zhan, Y.-T. Cheng, *J. Power Sources* **2019**, 410-411, 45; b) F. Schipper, M. Dixit, D. Kovacheva, M. Talianker, O. Haik, J. Grinblat, E. M. Erickson, C. Ghanty, D. T. Major, B. Markovsky, D. Aurbach, *J. Mater. Chem. A* **2016**, 4, 16073.

[2] a) T. Chen, X. Li, H. Wang, X. Yan, L. Wang, B. Deng, W. Ge, M. Qu, *J. Power Sources* **2018**, 374, 1; b) L. Pan, Y. Xia, B. Qiu, H. Zhao, H. Guo, K. Jia, Q. Gu, Z. Liu, *J. Power Sources* **2016**, 327, 273.

[3] K. Min, S. W. Seo, Y. Y. Song, H. S. Lee, E. Cho, *Physical Chemistry Chemical Physics Pccp* **2017**, 19, 1762.

[4] Y.-C. Li, W. Xiang, Z.-G. Wu, C.-L. Xu, Y.-D. Xu, Y. Xiao, Z.-G. Yang, C.-J. Wu, G.-P. Lv, X.-D. Guo, *Electrochim. Acta* **2018**, 291, 84.

[5] Y. Su, Y. Yang, L. Chen, Y. Lu, L. Bao, G. Chen, Z. Yang, Q. Zhang, J. Wang, R. Chen, S. Chen, F. Wu, *Electrochim. Acta* **2018**, 292, 217.

[6] L. Wu, X. Tang, X. Chen, Z. Rong, W. Dang, Y. Wang, X. Li, L. Huang, Y. Zhang, *J. Power Sources* **2020**, 445, 227337.

[7] P. Kang-Joon, J. Hun-Gi, K. Liang-Yin, K. Payam, Y. C. S., S. Yang-Kook, *Adv.Energy.Mater* **2018**, 8, 1801202.

[8] Y. Huang, X. Liu, R. Yu, S. Cao, Y. Pei, Z. Luo, Q. Zhao, B. Chang, Y. Wang, X. Wang, *ACS Appl. Mater. Interfaces* **2019**, 11, 40022.
